# Integrating UPLC-Q-Orbitrap MS with serum pharmacochemistry network and experimental verification to explore the pharmacological mechanisms of *Cynanchi stauntonii* rhizoma et radix against sepsis-induced acute lung injury

**DOI:** 10.3389/fphar.2024.1261772

**Published:** 2024-03-22

**Authors:** Hejun Gao, Ziyi Yuan, Haoxuan Liang, Youtan Liu

**Affiliations:** ^1^ Department of Anesthesiology, Shenzhen Hospital, Southern Medical University, Shenzhen, China; ^2^ The Third School of Clinical Medicine, Southern Medical University, Guangzhou, China

**Keywords:** *Cynanchi stauntonii* rhizoma et radix (Csrer), acute lung injury, apoptosis, pharmacochemistry network, p53

## Abstract

**Introduction:** Patients with sepsis are at an incremental risk of acute lung injury (ALI). Baiqian, also known as *Cynanchi stauntonii* rhizoma et radix (Csrer), has anti-inflammatory properties and is traditionally used to treat cough and phlegm. This study aimed to demonstrate the multicomponent, multitarget, and multi-pathway regulatory molecular mechanisms of Csrer in treating lipopolysaccharide (LPS)-induced ALI.

**Methods:** The bioactive components of Csrer were identified by ultrahigh-performance liquid chromatography Q-Orbitrap mass spectrometry (UPLC-Q-Orbitrap MS). Active targets predicted from PharmMapper. DrugBank, OMIM, TTD, and GeneCards were used to identify potential targets related to ALI. Intersection genes were identified for Csrer against ALI. The PPI network was analysed to identify prime targets. GO and KEGG analyses were performed. A drug–compound–target–pathway–disease network was constructed. Molecular docking and simulations evaluated the binding free energy between key proteins and active compounds. The protective effect and mechanism of Csrer in ALI were verified using an ALI model in mice. Western blot, Immunohistochemistry and TUNEL staining evaluated the mechanisms of the pulmonary protective effects of Csrer.

**Results:** Forty-six bioactive components, one hundred and ninety-two potential cross-targets against ALI and ten core genes were identified. According to GO and KEGG analyses, the PI3K-Akt, apoptosis and p53 pathways are predominantly involved in the “Csrer–ALI” network. According to molecular docking and dynamics simulations, ten key genes were firmly bound by the principal active components of Csrer. The “Csrer–ALI” network was revealed to be mediated by the p53-mediated apoptosis and inflammatory pathways in animal experiments.

**Conclusion:** Csrer is a reliable source for ALI treatment based on its practical components, potential targets and pathways.

## 1 Introduction

ALI and acute respiratory distress syndrome (ARDS) are common clinical syndromes associated with bacterial and viral infection, sepsis, and other diseases. ALI has an annual mortality rate of 4% among all hospitalized patients and accounts for 10% of all intensive care unit (ICU) inpatients (Duan and Liu, 2022). ARDS is distinguished by acute and progressive worsening of dyspnea, pulmonary edema and refractory hypoxemia (Thompson et al., 2017). ALI/ARDS caused by various risk factors have a familiar pathophysiological basis: overactivation of immune cells, “cytokine storm,” oxidative stress, hypoxia, inflammation and electrolyte disturbance (Sapru et al., 2015). A number of supportive therapies have been used for treating ALI/ARDS: surfactants, antioxidants, anticoagulants and neuromuscular blockers (Fan et al., 2018). However, previous treatment methods did not effectively reduce its mortality, highlighting the need for new treatment strategies (Witzenrath and Welte, 2022).

Traditional Chinese medicine (TCM) has a history of treating respiratory diseases for thousands of years (Yuan et al., 2022). ALI/ARDS, classified as “exogenous fever disease” in TCM, corresponds to “asthma syndrome,” “explosive asthma” and “puffing off” (Song et al., 2023). Baiqian, also known as *Cynanchum stauntonii* (Decne.) Schltr. ex Lévl. or *Cynanchi stauntonii* rhizoma et radix (Csrer), has been traditionally used to down-regulate qi, eliminate phlegm and relieve cough (Yue et al., 2014; Yu, 2017). However, the active components of Csrer and its mechanism of action against ALI still need to be fully understood. Therefore, bioinformatics analysis and animal experiments were utilized to prove the latent targets and mechanisms of Csrer in treating ALI.

**FIGURE 1:**
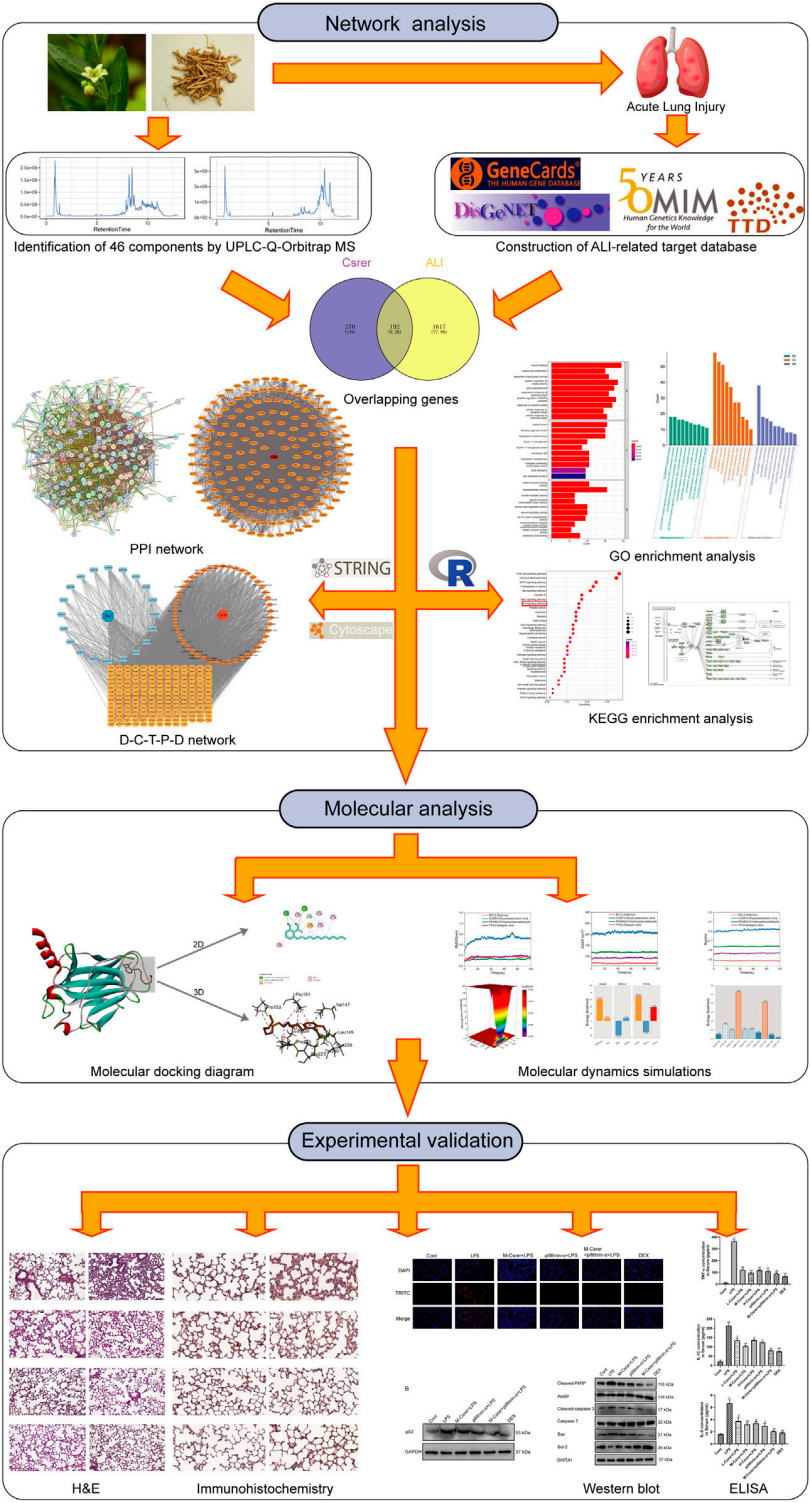
Workflow of the network pharmacological study of Csrer in treating ALI.

TCM contains complex compounds that activating multiple targets and pathways (Tong et al., 2021; Yang et al., 2021; Wang et al., 2022). Network analysis is a new method to elucidate complex pathophysiological processes by evaluating the interactions among TCM, components, target genes and diseases, to help understand the intrinsic laws of TCM and reveal the multi-target and multi-component effects of TCM (Hopkins, 2007). Network analysis based on bioinformatics and systems biology has extensive applications in drug target recognition, active ingredient detection, mechanism of action research, preclinical efficacy research and safety assessment. Its integral and systematic features are concordant with the principle of TCM preparation, providing a new method and strategy for the design and development of new drugs (Wang et al., 2020). UPLC-Q-Orbitrap MS, an emerging technology, was mainly used for the rapid identification of chemical components of TCM with high distinguishability and low interference (Ning et al., 2022). In this work, the main active components of Csrer were analysed by UPLC-Q-Orbitrap MS. Network analysis was then used to predict targets and pathways. Moreover, molecular docking and simulations can predict ligand–receptor interactions and binding capacity (Tong et al., 2021; Hou et al., 2022; Wang et al., 2022; Xia et al., 2022). Furthermore, we verified the pharmacologic and mechanism of Csrer on LPS-induced ALI/ARDS in mice experiments. The results show that Csrer has an apparent protective effect in treating ALI, which is expected to be an effective treatment to alleviate ALI. Figure 1 summarises the methodologies of this study.

## 2 Materials and methods

### 2.1 Preparation of Csrer sample

Csrer extract was obtained from Efong Pharmaceutical (Guangdong Province, China). According to the company’s quality inspection results, each 1 g of Csrer formula powder is equivalent to 4 g of Chinese herbal medicine. According to Chinese medicine classics, the dosage of decocting for adults is 5 g/d (equivalent to 1.25 g/d for powder), and the adult weight is 83 mg/(kg*d) (20.83 mg/(kg*d) for powder) according to 60 kg standard. If the dosage of adult decoction is 5 g/d, then the corresponding concentration of Csrer taken by mice is 9.01*20.83 = 187.68 mg/(kg*d) calculated with the following formula: the dose of animal B =W* the dose of animal A (mg/kg) (W = 9.01). For effect verification, 93.85, 187.68, and 375.35 mg/(kg*d) Csrer concentrations were used in the experiment. The Csrer powder was sifted through 300 mesh sieve, sorted aseptically and confected in 0.5% solution to prepare Csrer suspension for intragastric administration.

### 2.2 UPLC-Q-Orbitrap-MS conditions

Briefly, 100 µL of blood samples were prepared in 1.5  mL EP tubes on ice and added with 400 µL of ice methanol solution, swirled for 30 s, stored at −20°C for 30 min and centrifuged for 15 min (20,000 rcf, 4°C). The supernatant was collected and centrifuged under the same conditions. The supernatant after two centrifugations was transferred to the sample vial for UPLC-Q-Orbitrap-MS detection.

Vanquish Flex UPLC (Thermo Fisher Scientific, Bremen, Germany) with UPLC column ACQUITY UPLC T3 (100 mm*2.1 mm, 1.8 µm, Waters, UK) was used for the analysis of Chinese medicine samples. The mobile phase system was composed of 5 mmol/L ammonium acetate +5 mmol/L acetic acid (A) and LC-MS acetonitrile (acetonitrile) (B). The flow rate was controlled at 0.35 mL/min, and the gradient program was 0–1 min, 1%B; 1–9.5 min, 1–99%B; 9.5–11.5 min, 99–99%B; 11.5–12 min, 99–1%B; and 12–15 min, 1%B. The column temperature was 35°C. Q-Exactive high-resolution mass spectrometer (Thermo Fisher Scientific, Bremen, Germany) was used to acquire positive and negative ion mode mass spectrometry data. The optimum conditions of ion source were as follows: source temperature, 350°C; sheath gas, 12 Arb; spray voltage, 3.80 kV (positive ion mode), −3.50 kV (negative ion mode); and auxiliary gas, 45 Arb.

### 2.3 Prediction of the potential targets of the identified constituents in Csrer

The potential targets of the main active ingredients of traditional Chinese medicine in mouse serum were identified using PharmMapper (http://www.lilab-ecust.cn/PharmMapper/). These ingredients were then used to search for target genes by utilizing 2D molecules downloaded from PubChem (Ding et al., 2022). The UniProt ID (https://www.uniprot.org/) was standardized with the gene symbol and duplicate Csrer targets were eliminated (Tian et al., 2020).

### 2.4 Identification of ALI-related targets

Several databases are available for ALI targets, including the Online Mendelian Inheritance in Man (OMIM, https://omim.org/), DisGeNet (DisGeNet, https://www.disgenet.org/home/), Therapeutic Target Database (TTD, https://db.idrblab.net/ttd/) and GeneCards (GeneCards, https://www.genecards.org/home). The GeneCards database was used with a correlation score ≥ 10 as the screening criterion (Yang et al., 2021). The target genes of ALI were obtained after removing duplicates.

### 2.5 Protein-protein interaction (PPI) network construction

The VENNY platform (https://bioinfogp.cnb.csic.es/tools/venny/index.html) was used to determine cross-target genes. PPI networks were constructed with medium confidence of 0.400 on the STRING platform version 11.0 (https://string-db.org) (Xiang et al., 2022).

### 2.6 Function and pathway enrichment analyses

The R software and Bioconductor software package were used for Gene Ontology (GO) and Kyoto Encyclopedia of Genes and Genomes (KEGG) pathway enrichment analyses of cross-target genes under *p* < 0.05 and *q* value < 0.05.

### 2.7 Drug–compound–target–pathway–disease (DCTPD) network

The cross-target genes were imported into DAVID Bioinformatics Resources (https://david.ncifcrf.gov/search.jsp) for the KEGG pathway enrichment analyses to obtain the top 20 core pathways. Enrichment factor > 1.5 and a cutoff of *p* < 0.05 were applied (Bai et al., 2021; Ding et al., 2021). The top 20 core pathways were inputs for the KEGG pathway enrichment analysis. The drugs, active compounds, crossover genes, core pathways and diseases were analyzed in five aspects by using Cytoscape software version 3.9.0 to establish the DCTPD network model (Ding et al., 2021).

### 2.8 Molecular docking of compound–target interactions

The 3D structures of active ingredients, such as glabrone, p-hydroxybenzaldehyde, ginkgolic acid and dihydroartemisinic acid, were obtained from the PubChem database and the Protein Data Bank (https://www.rcsb.org/). OpenBabel was used for format conversion. AutoDock Tools version 1.5.7 was used to perform molecular docking. Discovery Studio 2021 was used to visually analyse the docking results (Yang et al., 2022b).

### 2.9 Molecular dynamic (MD) simulations

The molecular dynamics simulations of complexes involving Ginkgolic acid-Tp53, Dihydroartemisinic acid-CASP3, p-hydroxybenzaldehyde-PPARG, and Glabrone-BCL2 were performed using the Gromacs2022 software. Charmm36 was chosen as the protein force field, Gaff2 as the coordination force field (Van Der Spoel et al., 2005; Huang and MacKerell, 2013). The complexes were solvated in a cubic box with periodic boundary conditions of 1.2 nm, using the TIP3 water molecular model. Additionally, sodium and chloride ions were introduced to neutralize the charges of the solution system, aiming to replicate the real experimental environment as accurately as possible. Prior to the formal kinetic simulation, the complex underwent an initial minimization process of 50,000 steps utilizing a conjugate gradient algorithm. Subsequently, it was further equilibrated through canonical ensemble (NVT) and isothermal-isobaric ensemble (NPT) (310 K, 1 standard atmosphere) for a duration of 100 ps. Finally, molecular dynamics simulations were conducted at ambient temperature and pressure for a total duration of 100 ns.

### 2.10 Animals

Male C57BL/6 mice (10 weeks, 30 g) were purchased from the Guangdong Experimental Animal Center. The Medical Ethics Committee of Shenzhen Hospital of Southern Medical University (No. 2022-0281) provided ethical approval for the animal experiments. The mice were divided into eight groups and intragastrically administered with three concentrations of Csrer (93.85, 187.68, and 375.35 mg/kg) for 7 days. Dex (10 mg/kg/day) was used as the positive control and intraperitoneally injected for 7 days. On the 5th day, the p53 inhibitor (pifithrin-α, 3 mg/kg) was administered alone or combined with Csrer via intraperitoneal injection for 2 successive days. On the last day, LPS (4 mg/kg) was injected intratracheally, and the mice were sacrificed 24 h later (Figure 7A). Blood samples were collected from the mouse orbital venous plexus, centrifuged at 3,000 rpm for 15 min, then stored at −80°C. Collect lung tissue for subsequent experiments.

### 2.11 Histology

Lung tissue sections were stained with hematoxylin and eosin (H&E, 4 μm) and then evaluated for histopathological changes under light microscopy.

### 2.12 Determination of serum biochemical indicators

Blood samples were collected from the mouse orbital venous plexus. ELISA kits (Solarbio, Beijing, China) were used to determine the TNF-α, IL-1β, and IL-6 levels in serum.

### 2.13 Determination of biochemical indices of tissue homogenate

The MPO, ROS, SOD and MDA levels in the tissue homogenate were determined using detection kits (Nanjing Jiancheng Bioengineering Institute, Nanjing, China).

### 2.14 TUNEL staining

TUNEL staining was used to assess the apoptotic rate by the established protocol. The results are presented as the number of apoptotic cells per ×400 magnification field.

### 2.15 Western blot

The membranes were incubated with primary antibodies against p53, Bcl-2, Cleaved-caspase 3, PARP, Cleaved-PARP, Caspase 3, Bax, and GAPDH (Cell Signaling Technology, Danvers, MA, USA) overnight at 4°C. The protein bands were visualized using Super ECL Plus from Applygen Technologies, and their intensity was quantified using ImageJ software.

### 2.16 Immunohistochemistry

Lung sections (3 μm) were incubated with primary antibodies against p53 (1:100), Cleaved-PARP (1:100), Bax (1:100), and Cleaved-caspase 3 (1:100). On the second day, the slices were exposed to the corresponding secondary antibody for 1 hour and observed under a microscope.

### 2.17 Statistical analysis

All experiments were repeated three times and values are presented as the mean ± SD. One-way ANOVA followed by Tukey’s *post hoc* test was used to compare group differences. Statistical significance was set at *p* < 0.05. GraphPad Prism 8 software was used for all statistical analyses.

## 3 Results

### 3.1 Identification of active compounds and target genes in Csrer

**FIGURE 2:**
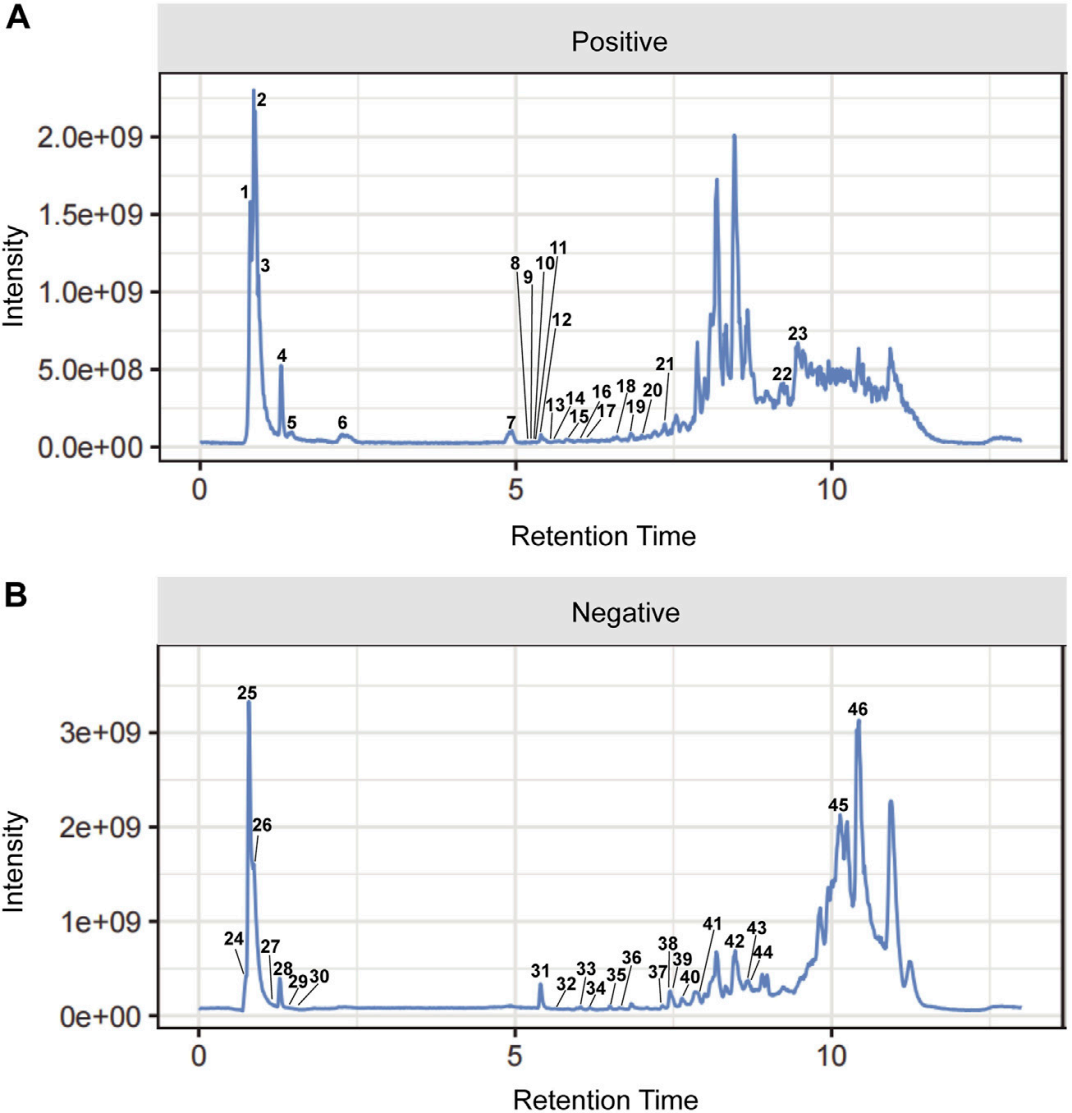
Total ion chromatograms (TICs) of serum pharmacochemistry of Csrer in ALI model mice. **(A)** Positive ion mode. **(B)** Negative ion mode.

**FIGURE 3:**
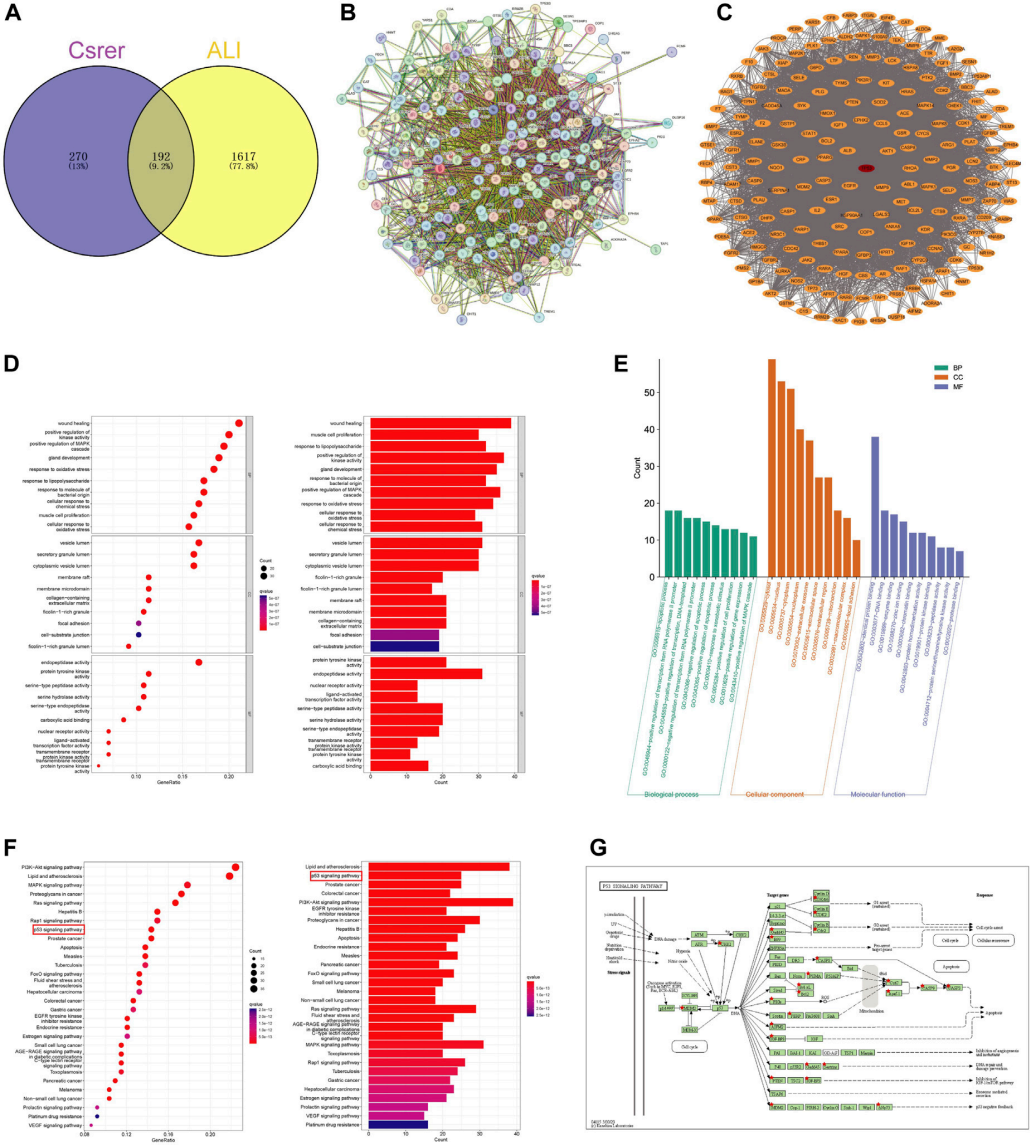
Identification targets of Csrer–ALI. **(A)** Venn diagram of intersecting targets of Csrer in treating ALI. **(B)** Primary PPI network. **(C)** PPI network. The red circle nodes represent core targets and the orange circle nodes represent noncore targets. **(D,E)** GO enrichment analysis. **(F)** KEGG enrichment analysis. **(G)** P53 signalling pathways of potential target genes of Csrer in treating ALI.

The UPLC-Q-Orbitrap MS with high sensitivity and accuracy was used to identify the components of Chinese medicine in serum. The chromatogram of positive and negative ions is shown in Figures 2A, B. Forty-six TCM monomer components were identified, and their detailed data including name, retention time and fragment ions are shown in Table 1. Among them, wogonoside, cimifugin, glabrone, 8-desoxygartanin and four other ingredients are flavonoids. Dihydroartemisinic acid, pristimerin and (+)-nootkatone are terpenoids. The remaining components include eleven alkaloids, twelve miscellaneous, five phenylpropanoids and other constituents. Meanwhile, the 2D molecular structures of these forty-six compounds were obtained from PubChem (Supplementary S1) and submitted to PharmMapper. 462 unique target genes of Csrer were acquired after deduplication. GeneCards, OMIM, DisGeNet and TTD were used to obtain 2033 ALI target genes, 1809 unique ALI target genes were identified after deduplication. The online mapping tool VENNY version 2.1.0 was used to intersect the potential targets of Csrer and ALI and 192 intersected targets were ultimately identified as candidate targets of Csrer treating ALI (Figure 3A).

**TABLE 1:** Identification of 46 chemical components absorbed into blood from Csrer by UPLC-Q-Orbitrap-MS.

No.	Rt (min)	Identified components	AdductedIon	CAS	Score
1	0.871	L (−)-Carnitine	[M + H]+	541–15–1	99.2
2	0.91	Trigonelline HCl	[M + H]+	6,138–41–6	85.2
3	0.915	2-Pyrrolidinecarboxylic acid	[M + H]+	147–85–3	92.7
4	1.438	Inosine	[M + H]+	58–63–9	76.2
5	1.455	Cytosine	[M + H]+	71–30–7	86.9
6	1.91	Nicotinamide	[M + H]+	98–92–0	93.9
7	4.599	Adenosine	[M + H]+	58–61–7	92.1
8	5.368	L-Leucine	[M + H]+	61–90–5	74
9	5.458	6-Hydroxyindole	[M + H]+	2,380–86–1	80.6
10	5.511	L-Phenylalanine	[M + H]+	63–91–2	73.8
11	5.511	Wogonoside	[M + H]+	51,059–44–0	90.1
12	5.569	4-Methyl-6,7-dihydroxycoumarin	[M + H]+	529–84–0	73.6
13	5.627	Adenine	[M + H]+	73–24–5	87
14	5.663	7-Hydroxycoumarin	[M + H]+	93–35–6	77.3
15	6	Cimifugin	[M + H]+	37,921–38–3	84.9
16	6.23	Glabrone	[M + H]+	60,008–02–8	78.8
17	6.387	7,8-Benzoflavone	[M + H]+	604–59–1	71.3
18	6.669	Ethyl 4-methoxycinnamate	[M + H]+	24,393–56–4	78.3
19	7.035	(+)-Nootkatone	[M + H]+	4,674–50–4	71.6
20	7.054	Sophoridine	[M + H]+	6,882–68–4	80.2
21	7.262	4-Methylumbelliferone	[M + H]+	90–33–5	77.7
22	8.823	alpha-Linolenic acid	[M + H]+	463–40–1	83.6
23	8.96	Corynoxeine	[M + H]+	630–94–4	74
24	0.843	Taurine	[M−H]−	107–35–7	93
25	0.902	Allantoin	[M−H]−	97–59–6	86.7
26	0.931	L-Tyrosine	[M−H]−	60–18–4	95.6
27	1.027	p-Hydroxybenzaldehyde	[M−H]−	123–08–0	82.8
28	1.182	Fumaric acid	[M−H]−	110–17–8	79.2
29	1.282	Uridine	[M−H]−	58–96–8	86.3
30	1.378	Calcium pantothenate	[M−H]−	137–08–6	84.9
31	5.614	Protocatechuic acid	[M−H]−	99–50–3	85.6
32	5.844	p-Coumaric acid	[M−H]−	501–98–4	88.3
33	6.141	Azelaic acid	[M−H]−	123–99–9	87.8
34	6.243	Vanillin	[M−H]−	121–33–5	93.1
35	6.558	Daidzein	[M−H]−	486–66–8	86.9
36	6.789	Tauroursodeoxycholic acid	[M−H]−	14,605–22–2	84.6
37	7.329	Cholic acid	[M−H]−	81–25–4	86
38	7.437	Hyodeoxycholic acid	[M−H]−	83–49–8	85.7
39	7.45	6-Gingerol	[M−H]−	23,513–14–6	90.9
40	7.837	Embelin	[M−H]−	550–24–3	76.4
41	8.098	Deoxycholic acid	[M−H]−	83–44–3	85.6
42	8.577	Ginkgolic acid (C13:0)	[M−H]−	20,261–38–5	75.8
43	8.796	Dihydroartemisinic acid	[M−H]−	85,031–59–0	85.2
44	8.831	Pristimerin	[M-H]-	1,258–84–0	73.1
45	10.25	8-Desoxygartanin	[M−H]−	33,390–41–9	72
46	11.639	Citric acid	[M−H]−	77–92–9	97.3

### 3.2 PPI analysis

STRING was used to construct a PPI network (Figure 3B). Disconnected nodes were removed, resulting in 192 nodes and 6,670 edges. The targets were ranked based on betweenness and centrality to identify the hub proteins. The core targets in the central PPI network included Tp53, ALB, AKT1, MMP9, EGFR, ESR1, CASP3, PPARG, HSP90AA1, and BCL2 (Figure 3C). These hub genes were further utilized in the molecular docking and molecular dynamic simulation studies.

### 3.3 GO function enrichment analysis

The R software with the Bioconductor package was used for the GO analysis of the 192 overlapping targets. The significantly enriched biological processes were identified and presented in Figures 3D, E; Supplementary S2. The enriched genes were mainly associated with disease damage, cell repair, and other related apoptotic processes, explaining the therapeutic potential of Csrer in treating ALI.

### 3.4 KEGG pathway enrichment analysis

The 192 cross-targets were subjected to KEGG pathway enrichment analysis using the DAVID database and the R software with Bioconductor package, and 154 candidate targets associated with various pathways were identified. The pathways are shown in Figure 3F; Supplementary S3. The major enriched KEGG pathways included the PI3K-AKT and p53 signaling pathways. Notably, the p53 signaling pathway exhibited a significant *q* value. Using the KEGG mapper platform, the specific positions of the 25 targets (red) related to the therapeutic effects in the p53 signaling pathway were plotted (Figure 3G). The findings suggest that Csrer may exert its therapeutic influence on ALI by modulating the p53 signaling pathway.

### 3.5 DCTPD network analysis

**FIGURE 4:**
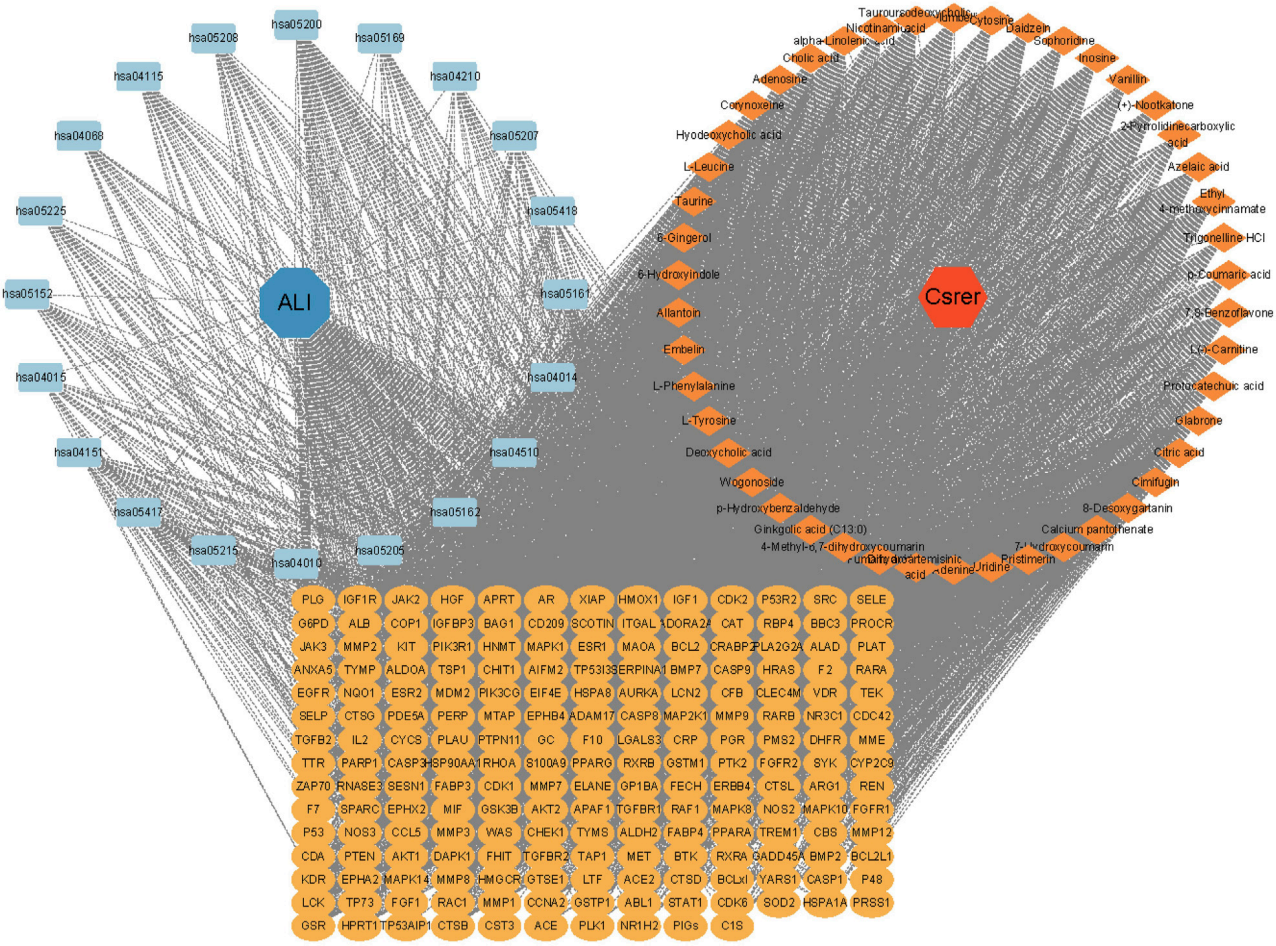
DCTPD network analysis.

Based on gene ratios and *q* values, the top 20 pathways were used to create a DCTPD network on Cytoscape version 3.9.0 (Figure 4). The diagram displayed the complicated relationship of Csrer in the treatment of ALI, including 260 total nodes (46 composite nodes, 192 target nodes, 20 core pathways, one Csrer node and one ALI node) and 4,908 edges. Nodes with different colors and shapes represented diseases, active components, targets, pathways and drugs. The number of lines linked with nodes indicated their significance in the network. The results revealed that the 46 main active compounds of Csrer were interconnected with 192 potential targets and 20 core pathways, indicating that the targets of active compounds were interconnected with the anti-ALI pathway. The network fully demonstrated the multi-component and multi-target intervention effect of Csrer in treating ALI.

### 3.6 Analysis of molecular docking results

**FIGURE 5:**
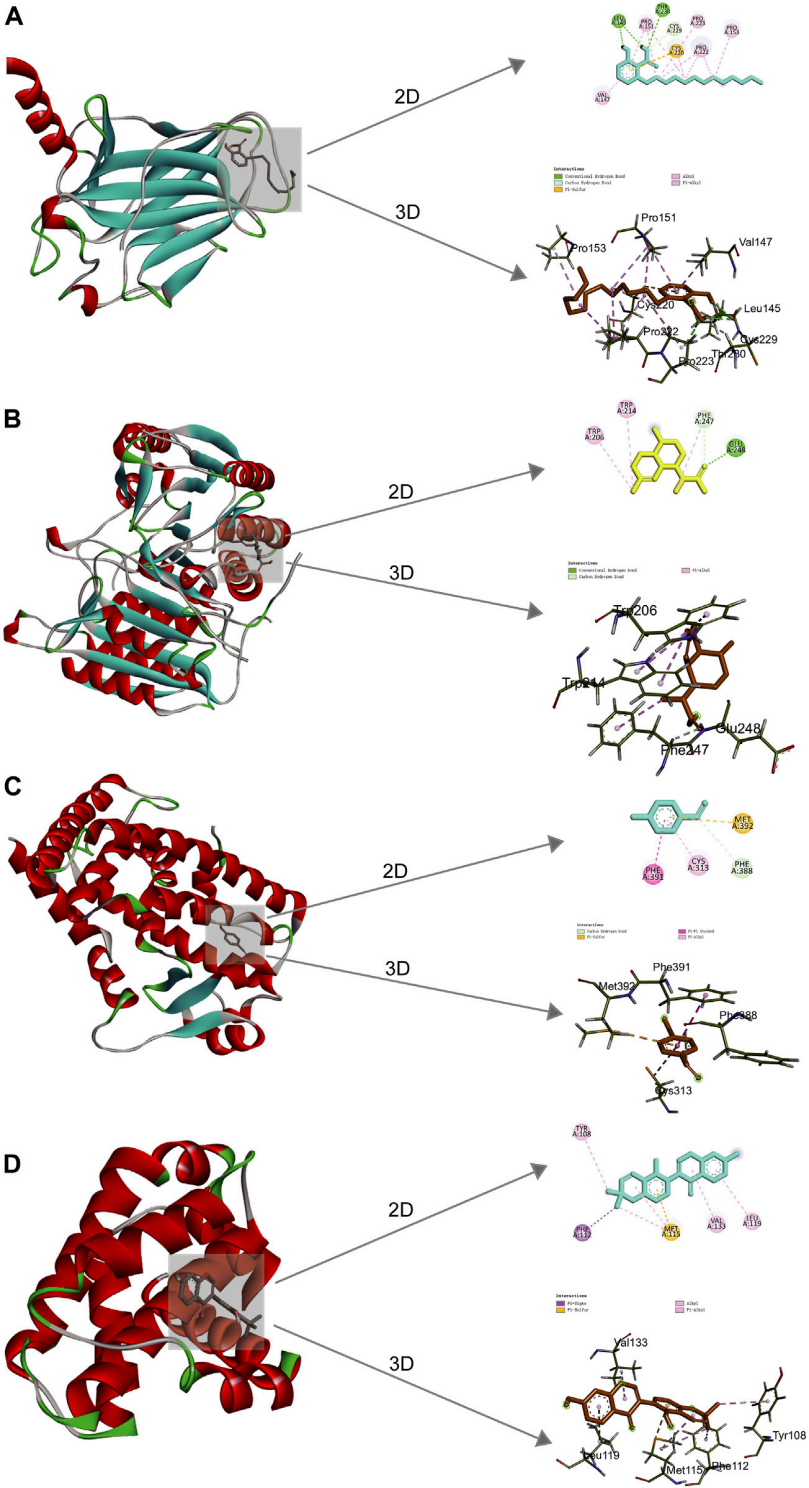
2D and 3D docking patterns of key targets and specific active compounds. **(A)** Ginkgolic acid binding to Tp53 crystal structure. **(B)** Dihydroartemisinic acid binding to CASP3 crystal structure. **(C)** p-hydroxybenzaldehyde binding to PPARG crystal structure. **(D)** Glabrone binding to BCL2 crystal structure.

Molecular docking was implemented to predict the potential therapeutic function of the main active components in Csrer to confirm the results of network pharmacology. Ten key targets, including Tp53, ALB, AKT1, MMP9, HSP90AA1, CASP3, EGFR, ESR1, PPARG, and BCL2, were selected based on the core targets in the PPI network and the targets involved in the p53 signaling pathway in KEGG. These proteins were docked with the four main active compounds glabrone, p-Hydroxybenzaldehyde, ginkgolic acid (C13:0), dihydroartemisinic acid. The docking affinity score was calculated using AutoDock, with a docking fraction of < −4.25 kcal/mol considered good docking affinity (Gaillard, 2018). The results showed that the main components and core proteins had good binding activity (Table 2). Furthermore, a few of 2D and 3D critical maps were generated (Figures 5A–D). The results showed that the common targets mentioned above had a docking affinity score to glabrone, p-Hydroxybenzaldehyde, ginkgolic acid (C13:0), dihydroartemisinic acid. Among them, the binding free energy of Tp53 and ginkgolic acid (C13:0) was −6.7 kcal/mol, which could be attributed to the hydrogen bonding with LEU-145, and THR-230 residue and the hydrophobic interactions with PRO-151, PRO-222, PRO-223, CYS-229 and CYS-220 residues. Similarly, CASP3 can form a stable complex with dihydroartemisinic acid. These findings explained the effectiveness of the four active components of Csrer in treating ALI. However, further animal experiments are necessary to validate the potential herbal compounds.

**TABLE 2:** Target molecule docking results for candidate active components.

Affinity (kcal/mol)										
Compound name	Tp53	ALB	AKT1	MMP9	EGFR	ESR1	CASP3	PPARG	HSP90AA1	BCL2
Glabrone	−8.8	−10.7	−10.5	−9.8	−8.5	−6.9	−8.1	−7.0	−9.1	−7.3
p-Hydroxybenzaldehyde	−5.8	−5.9	−5.4	−5.5	−4.7	−5.3	−4.7	−5.7	−5.9	−4.7
Ginkgolic acid (C13:0)	−6.7	−8.7	−8.1	−7.5	−5.6	−7.3	−6.2	−8.1	−7.8	−6.2
Dihydroartemisinic acid	−6.8	−7.9	−7.5	−8.4	−7.1	−8.8	−5.9	−7.3	−7.8	−6.9

### 3.7 Molecular dynamic simulations

**FIGURE 6:**
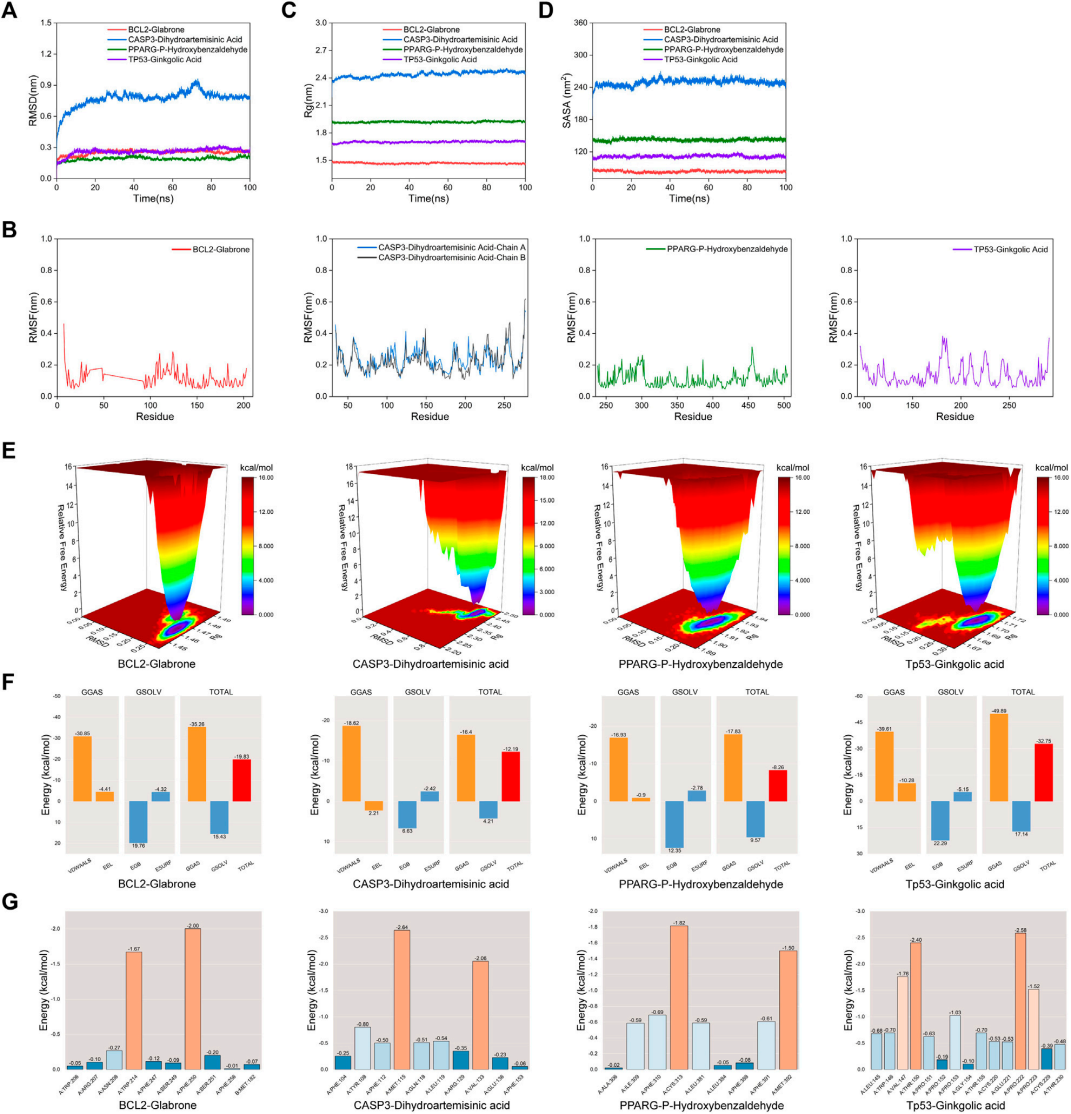
**(A)** RMSD, **(B)** RMSF, **(C)** Rg, **(D)** SASA, **(E)** FEL, **(F)** Combining free energy and **(G)** Energy contribution of amino acid residues plot of selected receptor–ligand complexes during molecular dynamic simulations.

Molecular Dynamic (MD) simulations were performed on complexes involving BCL2-Glabrone, CASP3-Dihydroartemisinic Acid, PPARG-P-Hydroxybenzaldehyde, and TP53-Ginkgolic Acid. The Root Mean Square Deviation (RMSD) simulation curve was calculated over a period of 100 ns after system stability testing. A smoother RMSD curve indicates greater complex stability. Figure 6A shows that all four groups of complexes exhibited minimal fluctuations within a range of 1 nm without any significant variations. The Root Mean Square Fluctuation (RMSF) curve illustrates the extent of amino acid residue fluctuations in the proteins during kinetic simulations. In Figure 6B, the RMSF curves of the four complexes exhibit fluctuations within a range of 1 nm without any significant variations. The radius of gyration (Rg) is a measure of the tightness and stability of a structure. A larger Rg indicates that the system experienced expansion during the MD simulation, while a smaller Rg indicates that the system remained tight and stable. Figure 6C shows that the Rg curves of all four complexes remained stable throughout the process, with only slight fluctuations. The Solvent Accessible Surface Area (SASA) is a factor used to study protein structure folding and stability. Figure 6D shows that the SASA curves of the four complexes remain stable throughout the process with slight fluctuations. These results suggest that the four protein-ligand complexes are highly stable. The diagram of the free energy landscape (FEL) illustrates the conformation with the lowest energy during the MD simulation. If the interaction between proteins and ligands is weak or unstable, multiple rough surface minimum energy clusters may appear in the free energy landscape map. Conversely, strong and stable interactions can form near-single and smooth energy clusters in the potential energy distribution. The figure displays the minimum energy value as dark purple/blue spots, indicating the most stable structure; and the red/yellow spots represent unstable structures. The result shows good stability of the complexes (Figure 6E). After evaluating the composite system’s stability, we computed the MM/PBSA binding free energies of four complexes. The average binding free energies for these complexes are −19.83, −12.19, −8.26, and −32.75 kcal·mol−1, respectively. The result indicates that the binding levels of BCL2-Glabrone and TP53-Ginkgolic acid complexes are strong, while the binding levels of the other two groups are good (Figure 6F). Additionally, Based on the result of amino acid residues involved in the binding, PHE-250 and TRP-214 play a major role in the BCL2-Glabrone interaction, others and so on (Figure 6G).

### 3.8 Csrer alleviates LPS-induced ALI mice by inhibiting the overexpression of p53

**FIGURE 7:**
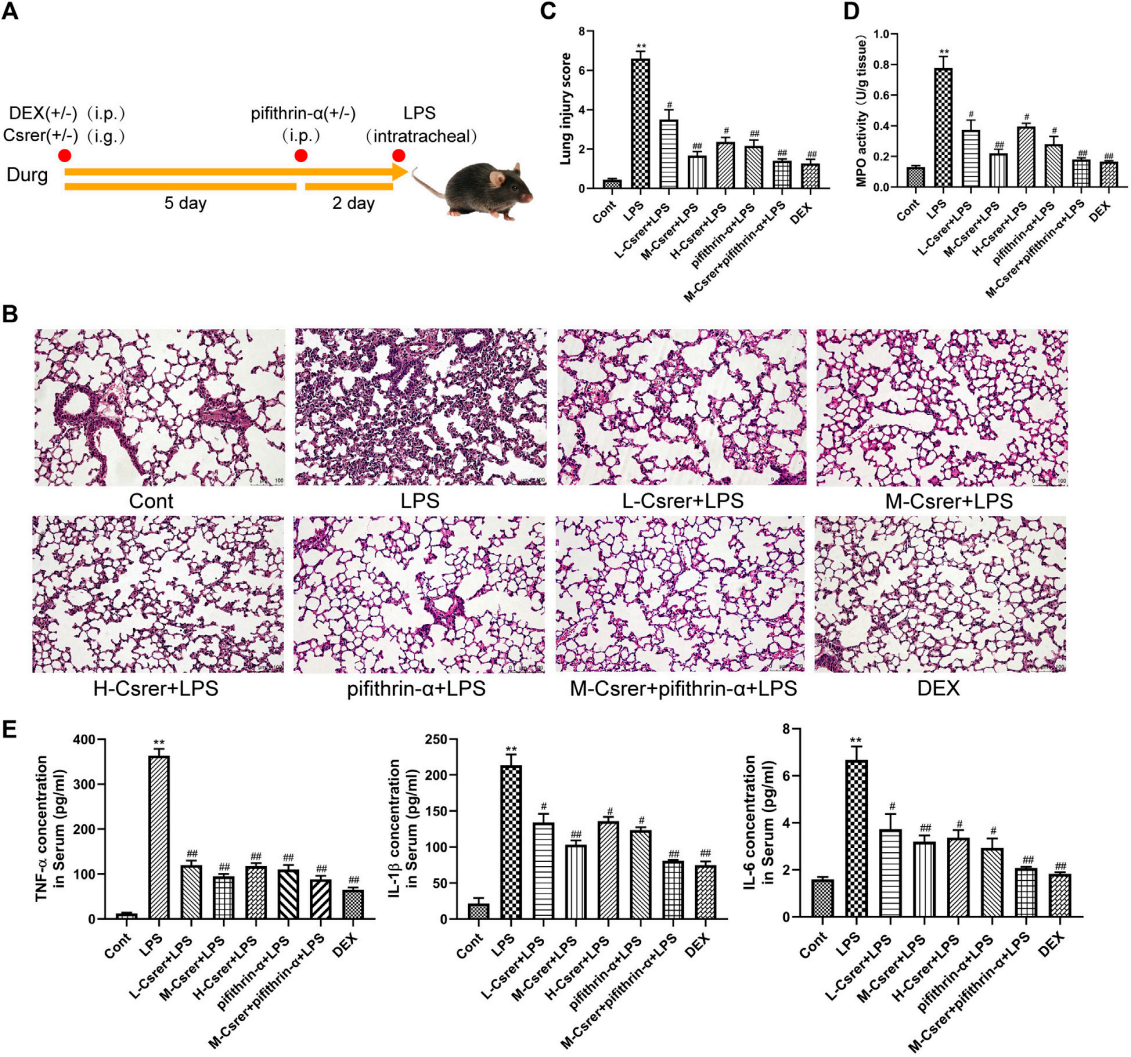
Csrer exerting protective effects on ALI mice by inhibiting the overexpression of p53. **(A)** Dosing time chart of Csrer treating ALI mice. **(B)** H&E-stained lung tissue L-Csrer, 93.85 mg/kg; M-Csrer, 187.68 mg/kg; H-Csrer, 375.35 mg/kg (×200 magnification). **(C)** Lung injury scores. **(D)** MPO activity levels. **(E)** Levels of TNF-α, IL-1β and IL-6 in serum. All data are shown as mean ± SD (*n* = 3). Compared with the control group, **p < 0.01 and *p < 0.05; compared with the model group, ^##^ *p* < 0.01 and ^#^ *p* < 0.05.

Lung sections of ALI model mice were analysed for pathological and inflammatory changes to evaluate the therapeutic effect of Csrer on LPS-induced ALI and confirm the involvement of the p53 signalling pathway predicted by the KEGG network. Pifithrin-α is a p53 inhibitor. Lung tissue was collected for H&E staining and MPO detection. After the LPS challenge, the alveolar walls of mice thickened, the alveolar and interstitial neutrophils increased and pulmonary capillaries congested. However, Csrer, pifithrin-α, Csrer + pifithrin-α and DEX significantly reduced the pathological changes, the lung injury score and the expression of MPO in the tissues of ALI model mice (Figures 7A–D). Considering the local expression of pro-inflammatory cytokines for the progression of ALI (Jiang et al., 2022), ELISA was used to determine the serum levels of TNF-α, IL-1β and IL-6. Csrer, pifithrin-α, Csrer + pifithrin-α and DEX can inhibit the up-regulation of TNF-α, IL-1β and IL-6 in mice with ALI (Figure 7E). These findings demonstrated that Csrer could inhibit the influx of inflammatory cells, pulmonary edema and LPS-induced cell damage and protect the integrity of the alveolar–vascular barrier. Therefore, Csrer can alleviate ALI injury by inhibiting the LPS-induced overexpression of p53.

### 3.9 Csrer alleviates ALI by inhibiting ROS-mediated the p53 protein overexpression

**FIGURE 8:**
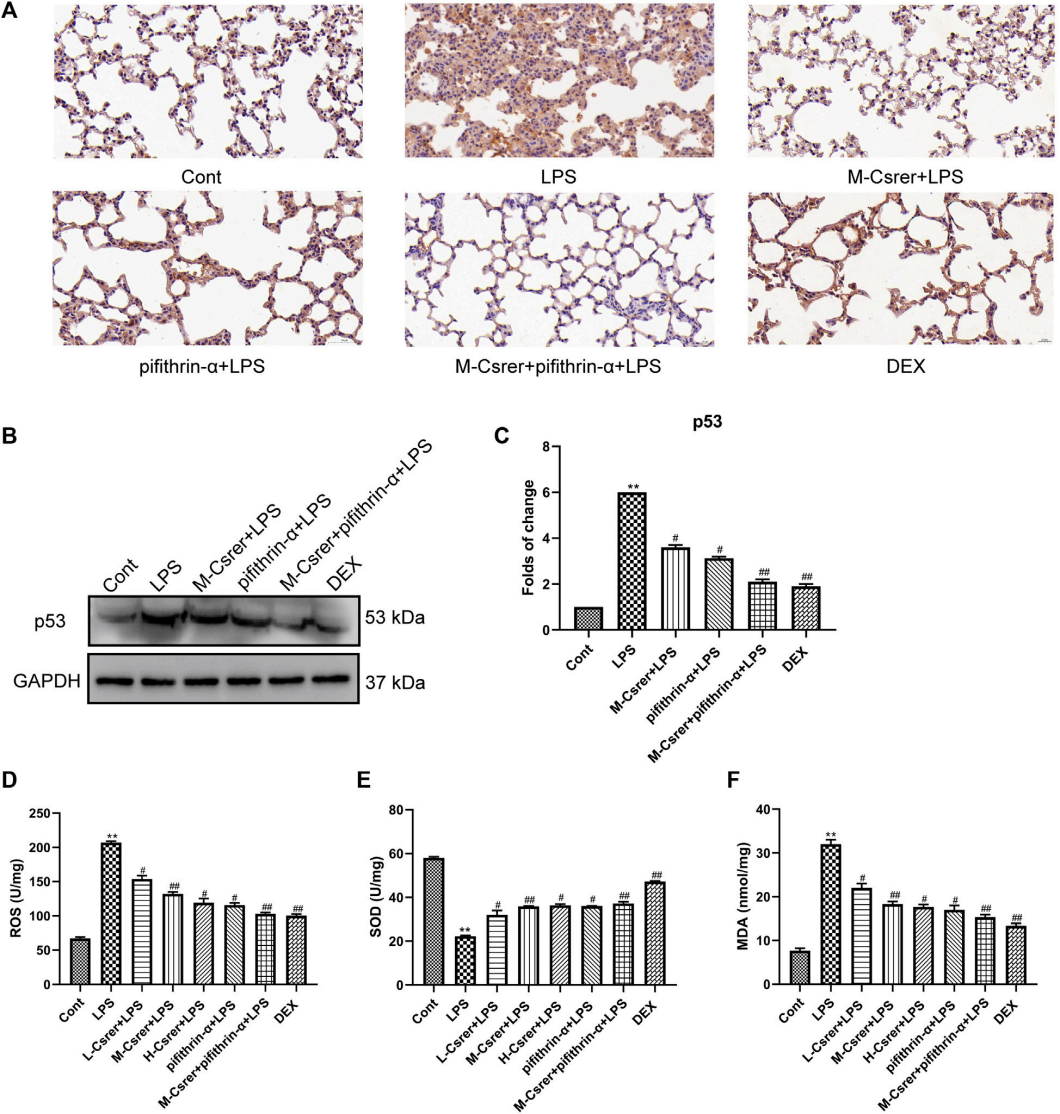
Csrer alleviation of ALI by inhibiting ROS-mediated the p53 protein overproduction. **(A)** Immunohistochemical staining of lung tissues for p53 markers (×400 magnification). **(B)** Expression of p53 in lung tissue. **(C)** Graphs representing the mean ± SD from three independent experiments. **(D–F)** Effect of Csrer on ROS, SOD and MDA levels in mouse lung tissues. All data are shown as the mean ± SD (*n* = 3). Compared with the control group, ***p < 0.001, **p < 0.01 and *p < 0.05; compared with the model group, ^##^ *p* < 0.01 and ^#^ *p* < 0.05.

Based on the p53 signaling pathway predicted by the KEGG enrichment analysis, the p53 protein was detected by IHC and Western blot. Our studies showed that LPS can promote p53 protein expression, whereas Csrer, pifithrin-α, Csrer + pifithrin-α or DEX can inhibit it (Figures 8A–C). Therefore, Csrer can alleviate ALI by inhibiting the LPS-induced overexpression of p53. Oxidative stress and ROS overexpression are essential for developing ALI (Zhang et al., 2021; Liang et al., 2022a). Based on previous studies, SOD and MDA are critical enzymes in maintaining ROS balance (Xia et al., 2022). In the present study, ELISA was used to detect the activities of ROS, SOD and MDA in lung tissue. The results revealed that the activities of ROS and MDA in the LPS-treated group increased, whereas that of SOD decreased. By contrast, Csrer, pifithrin-α, Csrer + pifithrin-α and DEX effectively inhibited the process (Figures 8D–F). These results indicated that Csrer could protect mice with ALI by inhibiting the p53 protein overexpression induced by ROS overproduction.

### 3.10 Csrer inhibits ALI through p53-mediated pulmonary epithelial cells apoptosis and inflammatory response in mice

**FIGURE 9:**
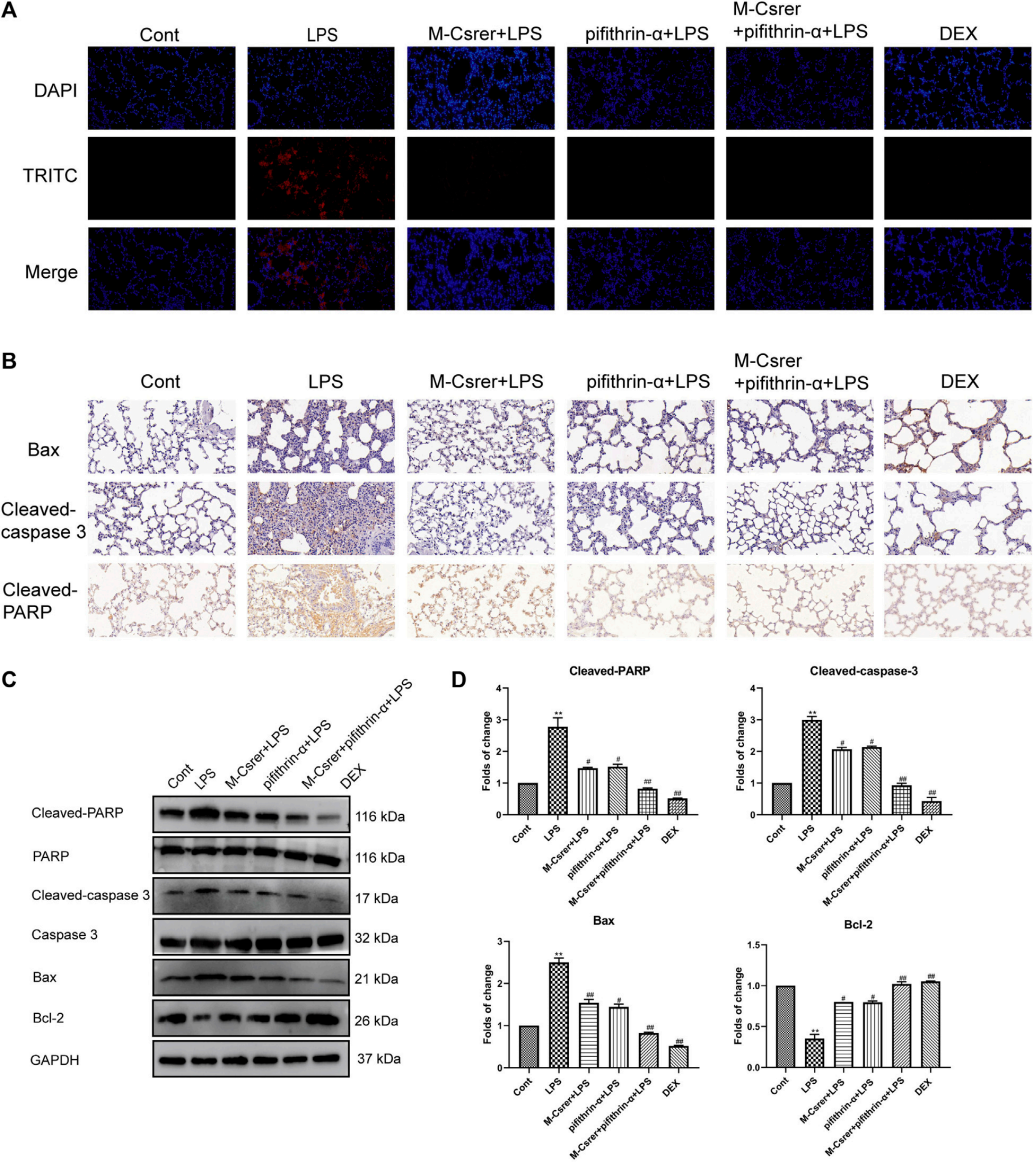
Csrer inhibits ALI through p53-mediated pulmonary epithelial cells apoptosis in ALI mice. **(A)** TUNEL staining (×400 magnification). **(B)** Immunohistochemical staining of lung tissues for Bax, Cleaved-caspase 3 and Cleaved-PARP markers (×400 magnification). **(C)** Expression of Cleaved-PARP, PARP, Cleaved-caspase 3, Caspase 3, Bax and Bcl-2 in lung tissue. **(D)** Graphs representing the mean ± SD from three independent experiments. Compared with the control group, ***p < 0.01 and **p < 0.01; compared with the model group, ^##^ *p* < 0.01 and ^#^ *p* < 0.05.

The p53 protein, a prime regulatory factor for cells to respond to multiple types of stress, including oxidative stress, can induce apoptosis (Yin et al., 2016; Liang et al., 2022a). In clarifying the protection mechanism of Csrer in LPS-induced ALI, great emphasis was laid on the association of p53 with the apoptotic pathway (Yang et al., 2022a). The number of apoptotic pulmonary epithelial cells after the LPS attack increased according to the TUNEL assay. Meanwhile, the LPS-induced apoptosis of pulmonary epithelial cells was significantly moderated with Csrer, pifithrin-α, Csrer + pifithrin-α and DEX (Figure 9A). LPS can promote the expression of Cleaved-PARP, Bax and Cleaved-caspase 3 but inhibit that of Bcl-2. By contrast, Csrer, pifithrin-α, Csrer + pifithrin-α and DEX can inhibit the expression of Cleaved-caspase3, Cleaved-PARP and Bax, and promote that of Bcl-2 (Figures 9B–D). Therefore, Csrer can reduce the apoptosis and inflammatory response of pulmonary epithelial cells by inhibiting the LPS-induced overexpression of p53.

## 4 Discussion

The modified Zhisou powder has cough-relieving effects. It can also improve coagulation function and regulate the levels of inflammatory factors (Dong et al., 2024). Csrer is one of the main components of this powder. Csrer is derived from the dry root and rhizome of Cynanchum stauntonii (Decne.) Schltr. ex Lévl. It contains steroidal saponins, triterpenes, flavonoids, acetophenone and its derivatives, sterols, lignans, and other compounds (Deng et al., 2017). Csrer is listed in the *Pharmacopoeia of the People’s Republic of China* (2020) and is known for its qi-reducing, phlegm-eliminating, and cough-relieving properties. Furthermore, studies demonstrated that Csrer has antitussive and expectorant effects (Ji et al., 2022). Some of the main active ingredients of Csrer play an anti-inflammatory role in protecting against ALI, such as vanillin, wogonoside and 4-methylumbelliferone. Studies of Csrer and its main active ingredients principally focused on a single targeted pathway or the mechanism of inflammation regulation. However, this method cannot thoroughly explain the whole therapeutical effect and mechanisms of Csrer for the treatment on ALI/ARDS. Therefore, further studies are demanded to elucidate the mechanism of Csrer against ALI.

ALI is a common condition caused by exogenous and endogenous factors that disrupt lung immune homeostasis, trigger systemic inflammation, and ultimately result in organ failure (Hu et al., 2020; Tao et al., 2023; Wu et al., 2023). A number of supportive therapies have been used for treating ALI/ARDS: surfactants, antioxidants, anticoagulants and neuromuscular blockers (Fan et al., 2018). However, previous treatment methods did not effectively reduce its mortality, highlighting the need for new treatment strategies (Witzenrath and Welte, 2022). Traditionally, Chinese medicine has been used for a long time. During the COVID-19 pandemic, TCM, including Csrer, has been certified to reduce coronavirus infection rates and effectively improve clinical symptoms. Baiqian, also known as *Cynanchum stauntonii* (Decne.) Schltr. ex Lévl. or *Cynanchi stauntonii* rhizoma et radix (Csrer), has been traditionally used to downregulate qi, eliminate phlegm and relieve cough (Yue et al., 2014; Yu, 2017). Csrer has many active ingredients, including vanillin, wogonoside and 4-methylumbelliferone. Previous studies found that vanillin defends lipopolysaccharide (LPS) -induced ALI by inhibiting NF-κB, p38 and ERK1/2 pathway (Guo et al., 2019). Wogonoside ameliorates LPS-induced ALI in mice (Zhang et al., 2014). Treating with the hyaluronic acid inhibitor 4-methylumbelliferone restrains LPS-induced pulmonary inflammation (McKallip et al., 2015). In this study, we use UPLC-Q-Orbitrap MS, pharmacochemistry network, molecular docking, molecular dynamics simulation and experimental *in vivo* to explain the whole therapeutical effect and mechanisms of Csrer in treating ALI/ARDS.

Forty-six active ingredients were identified using UPLC-Orbitrap Fusion MS, and 1809 ALI disease targets were obtained from four databases. Using VENNY, 192 potential targets for Csrer in treating ALI were identified. Subsequently, the PPI network analysis revealed that Tp53, ALB, AKT1, MMP9, EGFR, ESR1, CASP3, PPARG, HSP90AA1, and BCL2 are the core targets, with significant interactions between them. These core targets contained critical processes, such as anti-inflammatory responses, apoptosis, and pyroptosis. For example, overexpression of CARM1 may restrain the development of lung cancer by targeting Tp53 via regulating CTNNB1 (Hu et al., 2020). Therefore, these findings suggested that Csrer may exert its pharmacological effects through multiple targets and mechanisms.

The GO and KEGG enrichment analyses showed that multiple biological processes and signaling pathways directly contribute to the development and occurrence of “Csrer–ALI” thereby suggesting the mechanisms of Csrer in treating ALI. Among them, the p53 signaling pathway, which has great importance on inflammation and apoptosis, was selected to explore the mechanism of Csrer in treating ALI. A previous study showed that Hsp90 could alleviate endothelial cell dysfunction in ALI by promoting LPS-induced phosphorylation of MDM2, increasing p53 degradation, and reducing inflammatory cell apoptosis (Barabutis et al., 2015). The present study found that downregulating Tp53 expression can inhibit the p53 signaling pathway, thus reducing the inflammatory response and apoptosis.

The DCTPD network analysis revealed forty-six active components of Csrer for treating ALI. The results of molecular docking and molecular dynamic simulations between the compounds and targets further demonstrated the potential of Csrer as a drug for treating ALI. They confirmed the excellent binding of the active components with key proteins.

**FIGURE 10:**
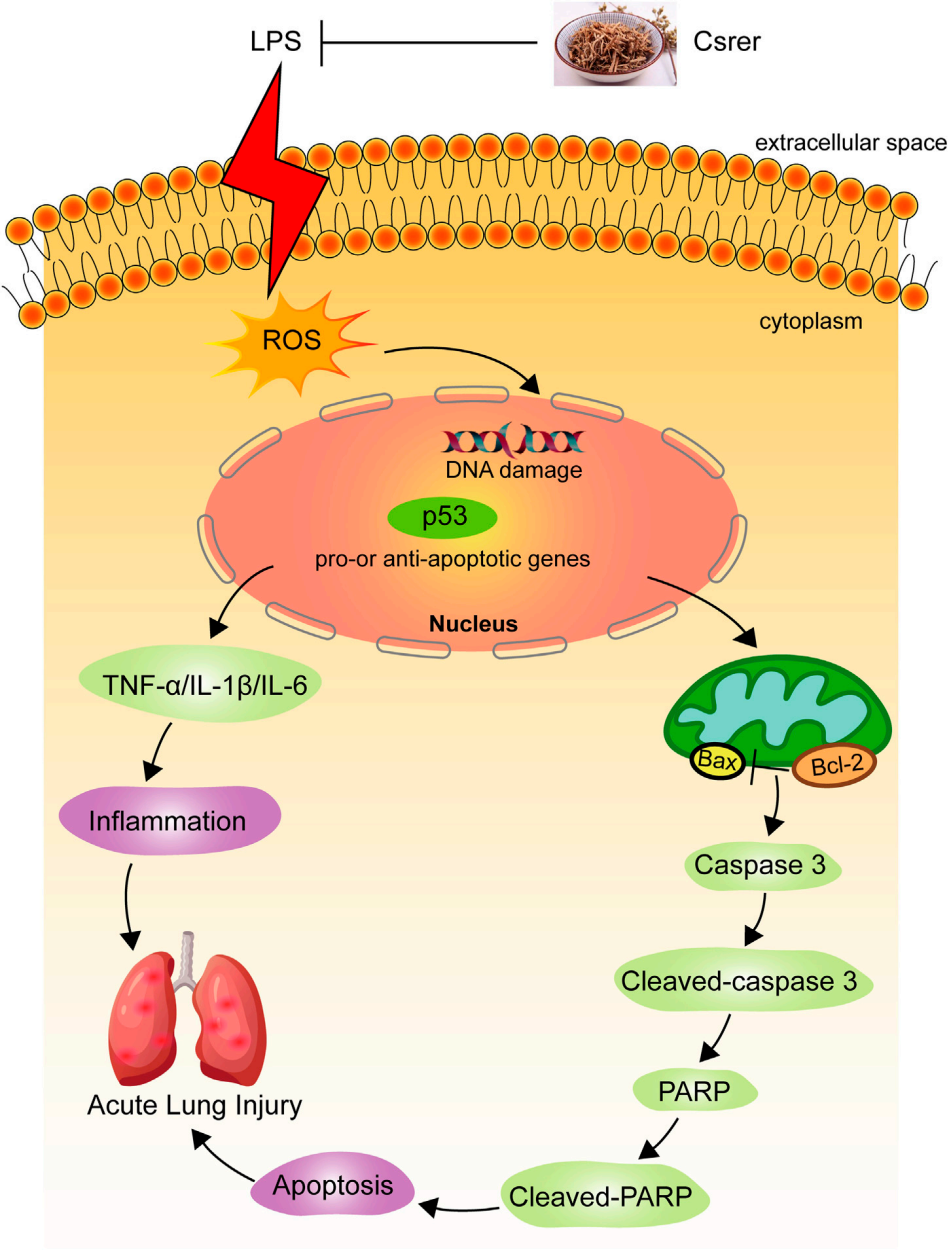
Mechanism of Csrer attenuating LPS-induced acute lung injury.

An LPS-induced ALI model was established to explore the remedial capacity of Csrer to sustain the findings of network analysis and molecular docking. The results found that LPS upregulated the expression of TNF-α, IL-1β and IL-6. Oxidative stress is an imbalance between the generation and destruction of free radicals; it is involved in the emergence and development of diseases that may cause organ damage (Song et al., 2014; Zhao et al., 2017; Zou et al., 2019). The endogenous ROS from the mitochondria is the uppermost measure of oxidative stress, playing a crucial role in many physiological and pathological settings (Dan Dunn et al., 2015). SOD, as an antioxidant enzyme, can accelerate the removal of free radicals and harmful peroxides, thereby protecting the structural and functional integrity of the cell membrane (Konishi et al., 2005; Shen et al., 2019). As the primary product of lipid peroxidation (Ayala et al., 2014), MDA is used to evaluate lipid peroxidation. In the present study, Csrer can downregulate the level of MDA while increasing SOD activities. Thus, the ability to remove ROS was enhanced, and the damages caused by oxidative stress were alleviated. Oxidative stress, a critical part of the cell damage process, has been identified as a core element in cell apoptosis (Liang et al., 2022b; Liu et al., 2022). Meanwhile, oxidative stress-induced apoptosis is closely connected with the p53 signaling pathway. With the activation of this pathway, oxidative stress eliminates the injury and death of cardiomyocytes by regulating the expression of apoptosis-related proteins (Zheng et al., 2020). LPS promotes the apoptosis of lung cells by increasing ROS and upregulating the expression of p53, as experimentally confirmed in the present study (Figure 10). Therefore, Csrer can alleviate ALI by inhibiting apoptosis of lung epithelial cells induced by ROS-mediated p53 overexpression.

Apoptosis maintains body balance by eliminating damaged and abnormal cells *in vivo* (Kędzierska et al., 2016). Bcl-2, Cleaved-PARP, Bax and Cleaved-caspase 3, as four essential proteins in the mitochondrion-mediated apoptotic pathways, are often used to evaluate the level of apoptosis. p53 is activated and combined with DNA when stimulated by DNA damage and oxidative stress, and it mediates apoptosis in the case of any irreparable DNA damage (Levine, 2020). In the present study, LPS activated endogenous apoptosis pathways by inhibiting the expression of Bcl-2 and upregulating those of p53, Bax, Cleaved-PARP and Cleaved-caspase 3. In general, Csrer can effectively suppress this process.

In summary, Csrer can alleviate ALI inflammatory response and apoptosis by inhibiting LPS-induced p53 overexpression. Through UPLC-Q-Orbitrap MS, network analysis and experimental verification, our study preliminarily clarified the serum material basis and mechanism of Csrer in ALI treatment, laying an experimental foundation for the clinical development and preparation of Csrer for treating ALI. Further studies including targeted metabolomic, proteomic and genomic are needed to understand the molecular mechanisms and related metabolic pathways of Csrer.

## Data Availability

The datasets presented in this study can be found in online repositories. The names of the repository/repositories and accession number(s) can be found in the article/Supplementary Material.

## Abbreviations

ALI, acute lung injury; ARDS, acute respiratory distress syndrome; BP, biological process; CC, cellular component; Csrer, *Cynanchum stauntonii (Decne.) Schltr. ex H.Lév. or Cynanchistauntonii Rhizoma Et Radix*; DAVID, database for annotation, visualization and integrated discovery; Dex, dexamethasone; DCTPD, drug–compound-target–pathway-disease; FEL, free energy landscape; GO, gene ontology; H&E, hematoxylin and eosin; KEGG, kyoto encyclopedia of genes and genomes; LPS, lipopolysaccharide; PARP, poly—(ADP)—ribose poly merase; MF, molecular function; PPI, protein-protein interaction; ROS, reactive oxygen species; SOD, Superoxide dismutase; TBST, TBS containing 0.1% tween; TUNEL, terminal deoxynucleotidyl transferase dUTP nick-end labeling; OMIM, the online mendelian inheritance in man database; PDB, the protein data bank; TTD, the therapeutic target database; TCM, traditional chinese medicine; RMSD, RToot mean square deviation; RMSF, The root mean square fluctuation; UPLC-Q-Orbitrap MS, ultrahigh-performance liquid chromatography Q-Orbitrap mass spectrometry; Rg, radius of gyration; SASA, solvent accessible surface area.
