# Supplementary material for: Integrating UPLC-Q-Orbitrap MS with serum pharmacochemistry network and experimental verification to explore the pharmacological mechanisms of Cynanchi stauntonii rhizoma et radix against sepsis-induced acute lung injury
Source: Front Pharmacol. 2024 Mar 22;15:1261772. doi: 10.3389/fphar.2024.1261772 (PMC10995315; doi:10.3389/fphar.2024.1261772)
Supplement: Supplementary file 1 [file DataSheet1.docx]

**Supplementary 1.**

Identification of 46 chemical components absorbed into blood from Csrer by UPLC-Q-Orbitrap MS.

| No. | Identified components | Ontology | Formula | Chemical structure |
| --- | --- | --- | --- | --- |
| 1 | L(-)-Carnitine | Alkaloids | C7H15NO3 | 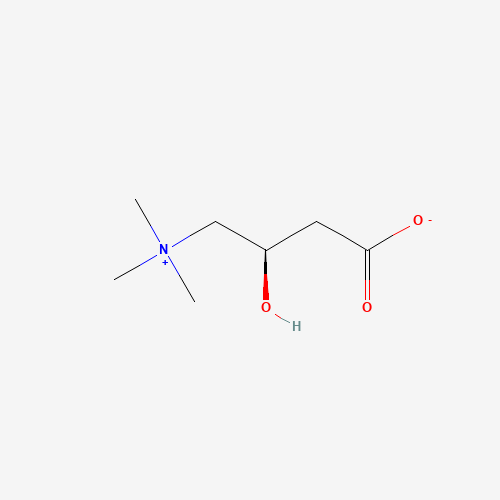 |
| 2 | Trigonelline HCl | Alkaloids | C7H7NO2.HCl | 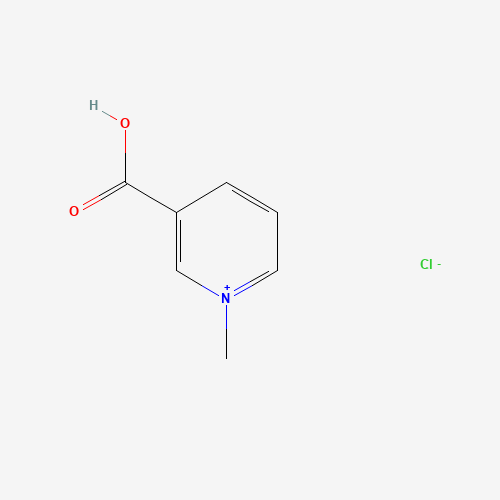 |
| 3 | 2-Pyrrolidinecarboxylic acid | Miscellaneous | C5H9NO2 | 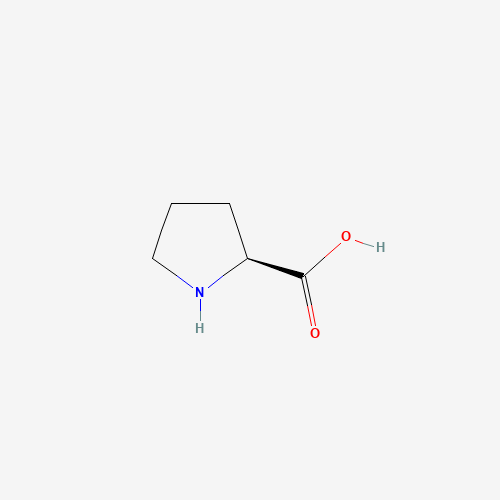 |
| 4 | Inosine | Alkaloids | C10H12N4O5 | 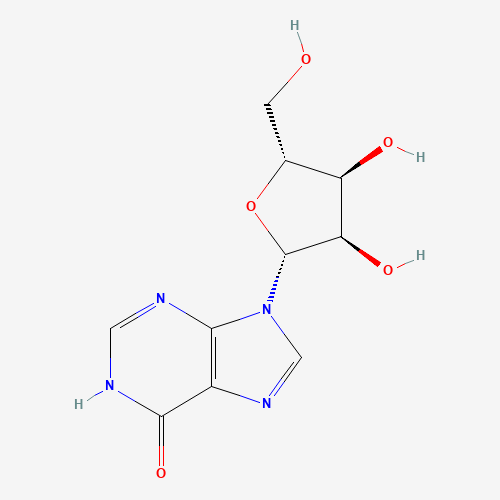 |
| 5 | Cytosine | Alkaloids | C4H5N3O | 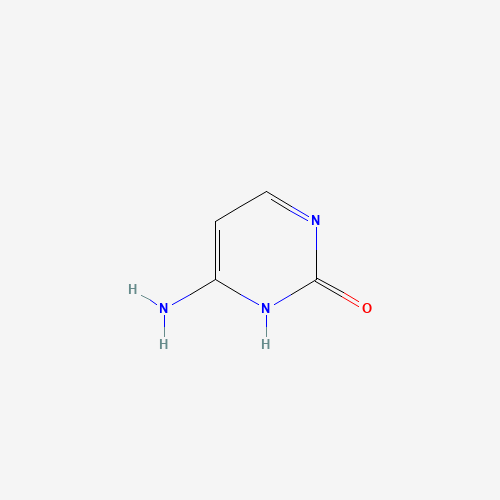 |
| 6 | Nicotinamide | Alkaloids | C6H6N2O | 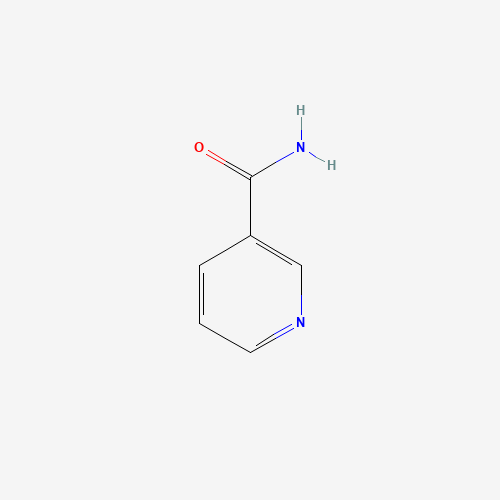 |
| 7 | Adenosine | Miscellaneous | C10H13N5O4 | 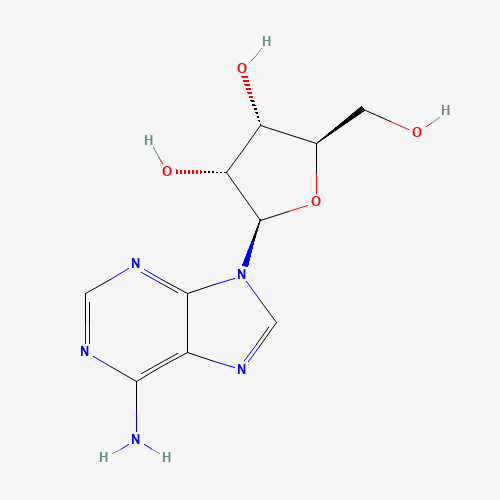 |
| 8 | L-Leucine | Miscellaneous | C6H13NO2 | 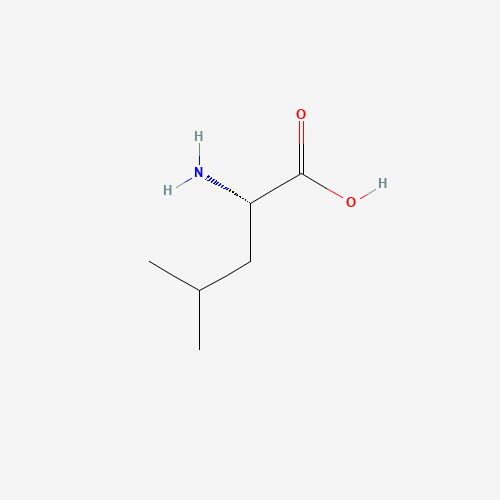 |
| 9 | 6-Hydroxyindole | Alkaloids | C8H7NO | 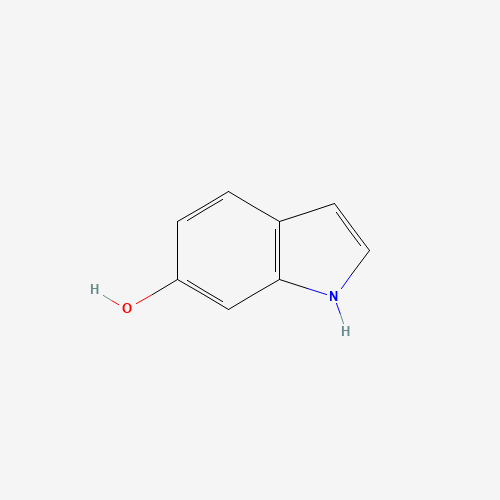 |
| 10 | L-Phenylalanine | Miscellaneous | C9H11NO2 | 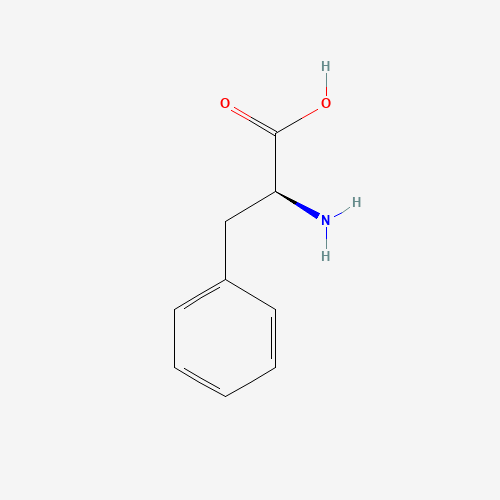 |
| 11 | Wogonoside | Flavonoids | C22H20O11 | 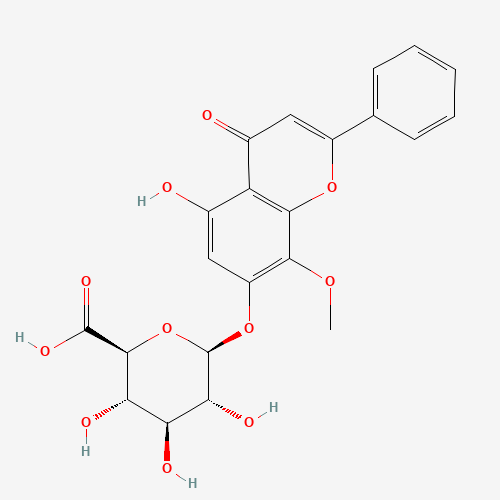 |
| 12 | 4-Methyl-6,7-dihydroxycoumarin | Phenylpropanoids | C10H8O4 | 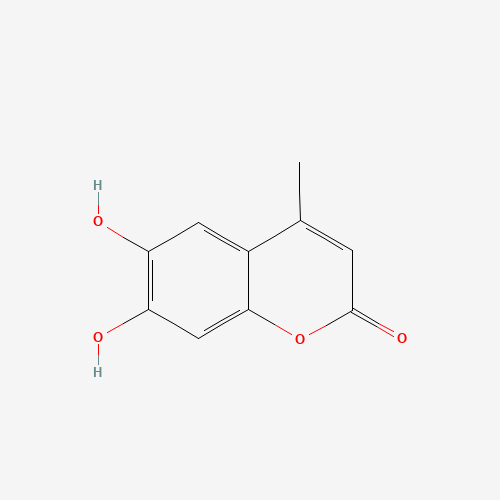 |
| 13 | Adenine | Alkaloids | C5H5N5 | 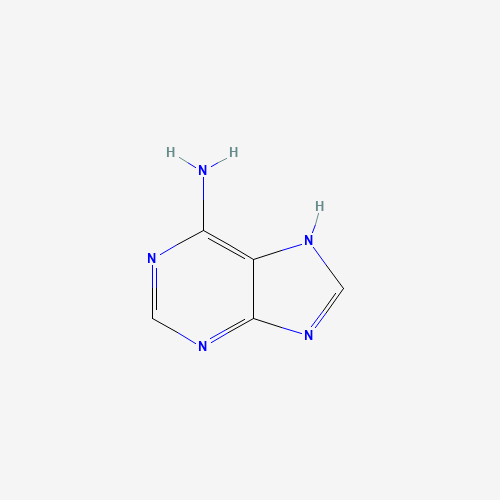 |
| 14 | 7-Hydroxycoumarin | Phenylpropanoids | C9H6O3 | 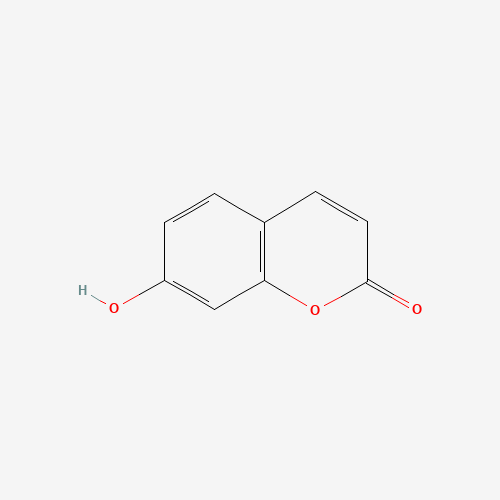 |
| 15 | Cimifugin | Flavonoids | C16H18O6 | 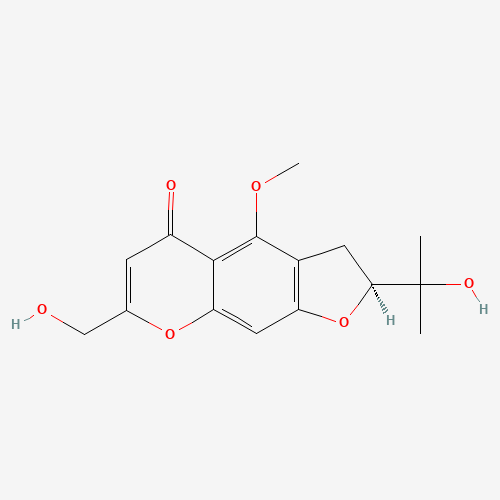 |
| 16 | Glabrone | Flavonoids | C20H16O5 | 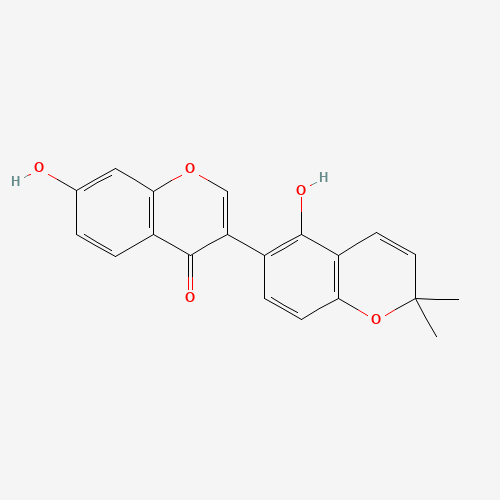 |
| 17 | 7,8-Benzoflavone | Flavonoids | C19H12O2 | 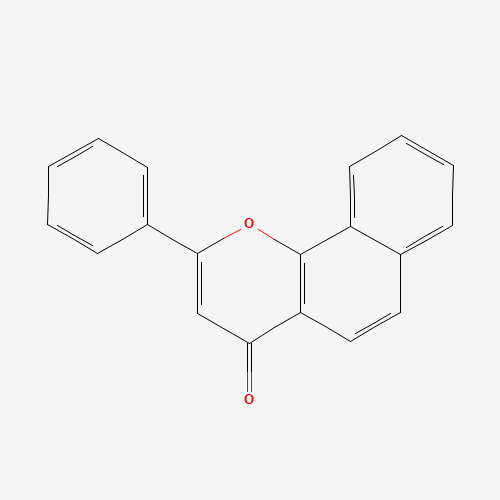 |
| 18 | Ethyl 4-methoxycinnamate | Phenylpropanoids | C12H14O3 | 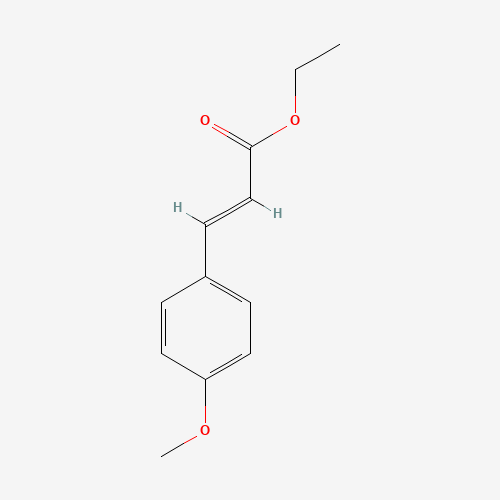 |
| 19 | (+)-Nootkatone | Terpenoids | C15H22O | 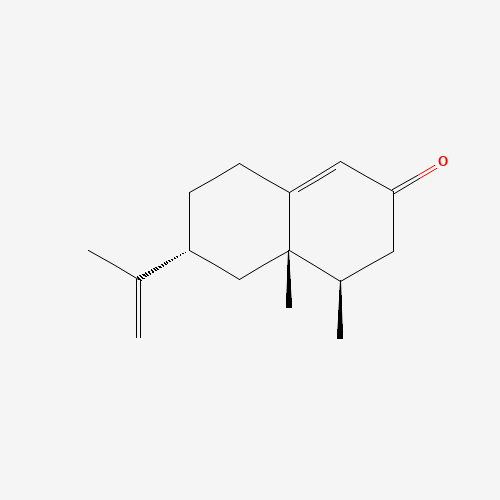 |
| 20 | Sophoridine | Alkaloids | C15H24N2O | 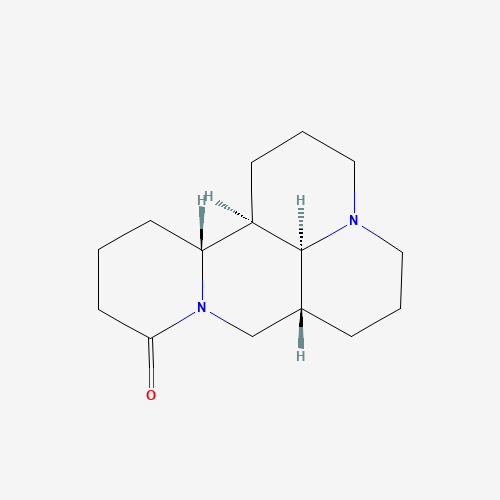 |
| 21 | 4-Methylumbelliferone | Phenylpropanoids | C10H8O3 | 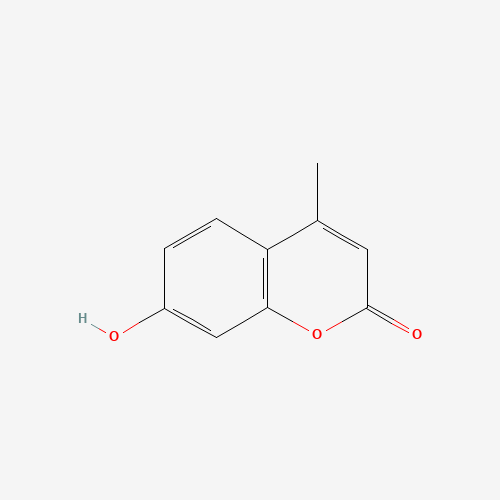 |
| 22 | alpha-Linolenic acid | Miscellaneous | C18H30O2 | 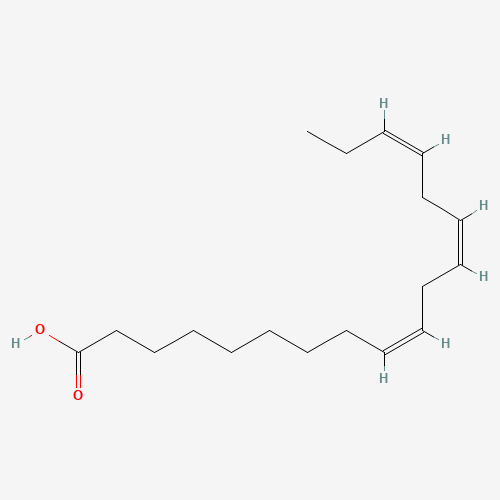 |
| 23 | Corynoxeine | Alkaloids | C22H26N2O4 | 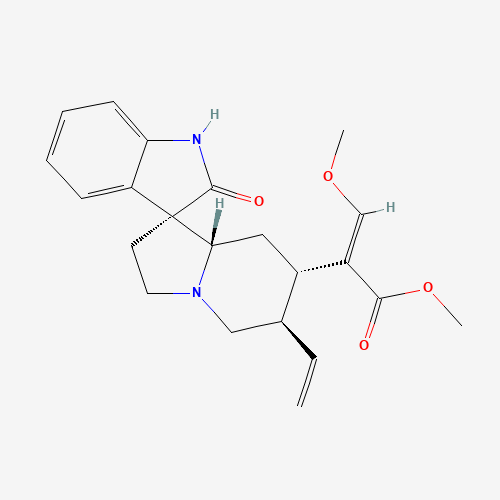 |
| 24 | Taurine | Miscellaneous | C2H7NO3S | 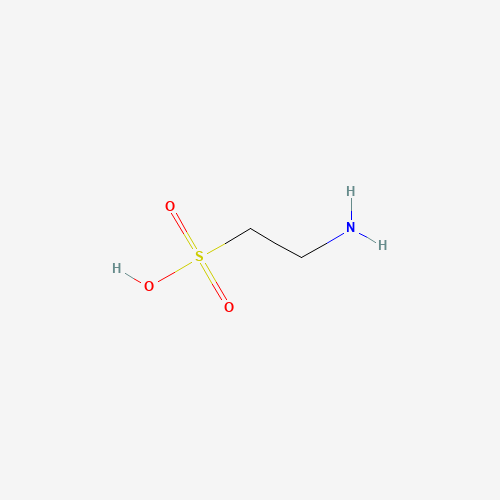 |
| 25 | Allantoin | Alkaloids | C4H6N4O3 | 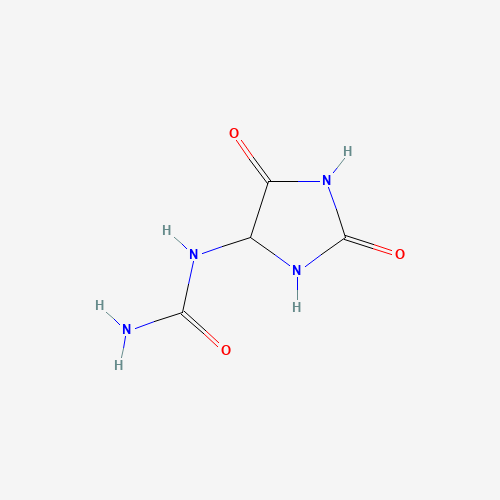 |
| 26 | L-Tyrosine | Miscellaneous | C9H11NO3 | 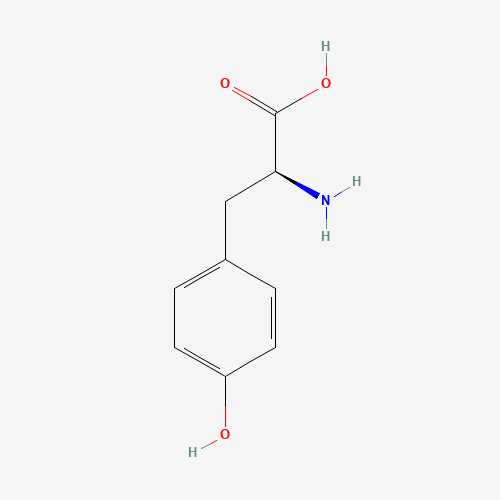 |
| 27 | p-Hydroxybenzaldehyde | Phenol | C7H6O2 | 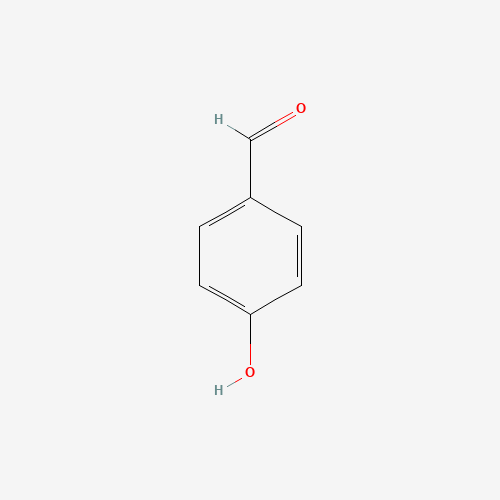 |
| 28 | Fumaric acid | Miscellaneous | C4H4O4 | 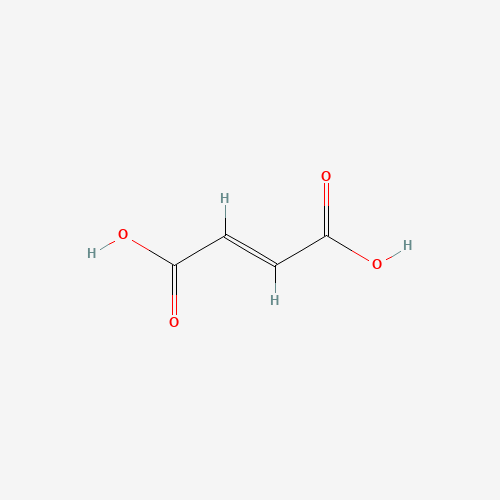 |
| 29 | Uridine | Miscellaneous | C9H12N2O6 | 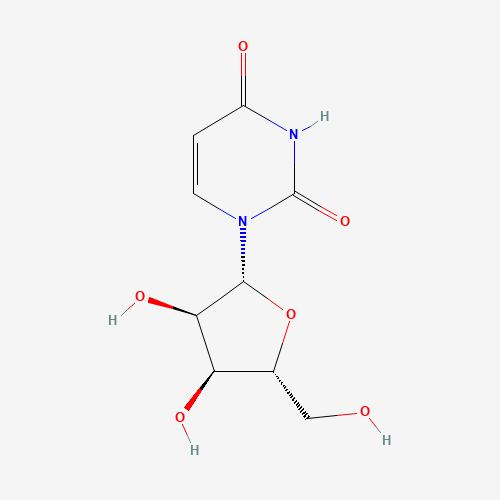 |
| 30 | Calcium pantothenate | Alkaloids | C9H17NO5.1/2 Ca | 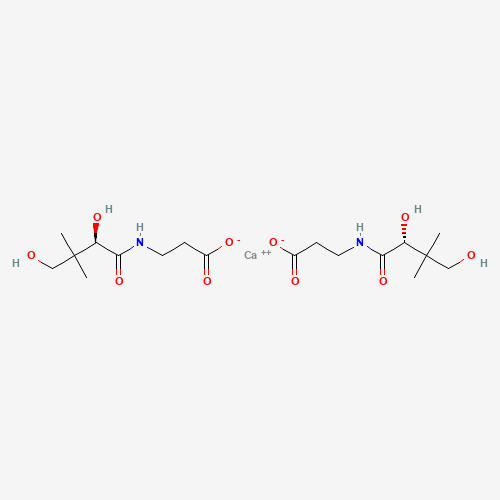 |
| 31 | Protocatechuic acid | Phenolic_acids | C7H6O4 | 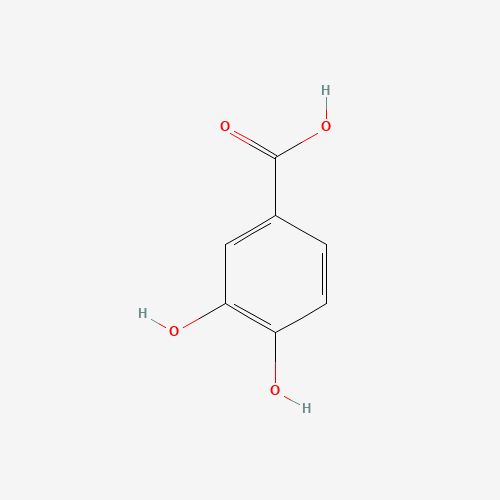 |
| 32 | p-Coumaric acid | Phenylpropanoids | C9H8O3 | 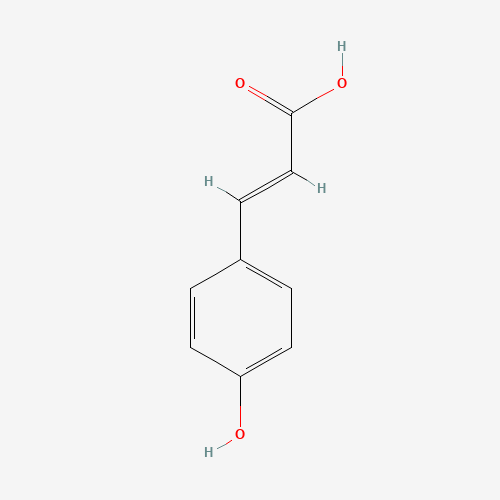 |
| 33 | Azelaic acid | Miscellaneous | C9H16O4 | 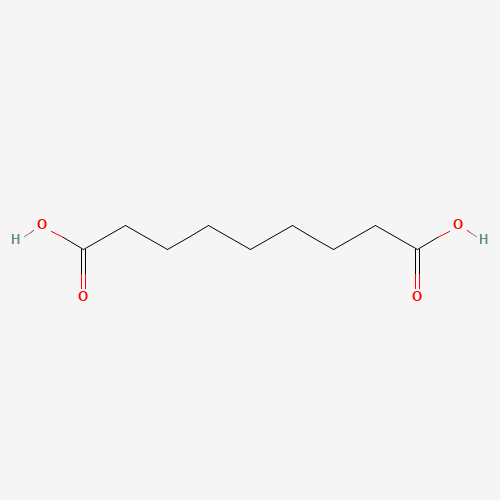 |
| 34 | Vanillin | Phenol | C8H8O3 | 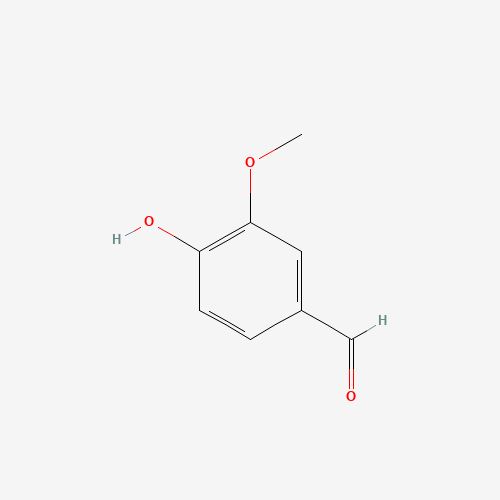 |
| 35 | Daidzein | Flavonoids | C15H10O4 | 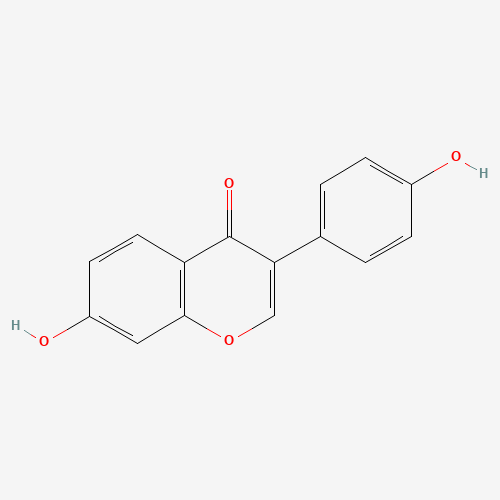 |
| 36 | Tauroursodeoxycholic acid | Steroids | C26H45NO6S | 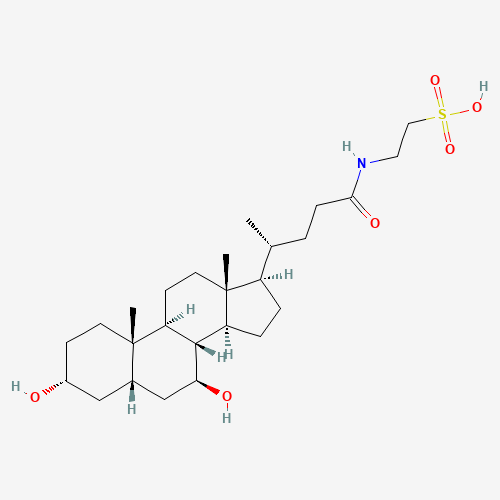 |
| 37 | Cholic acid | Steroids | C24H40O5 | 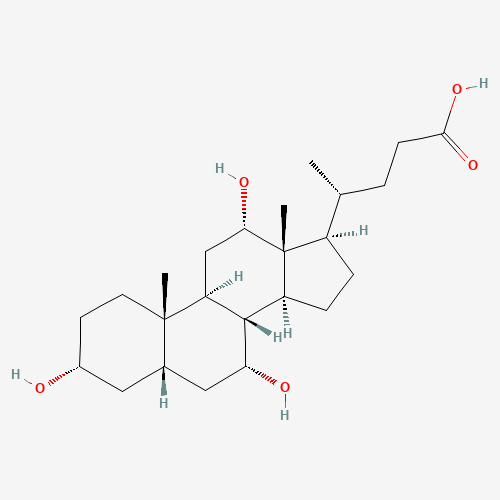 |
| 38 | Hyodeoxycholic acid | Steroids | C24H40O4 | 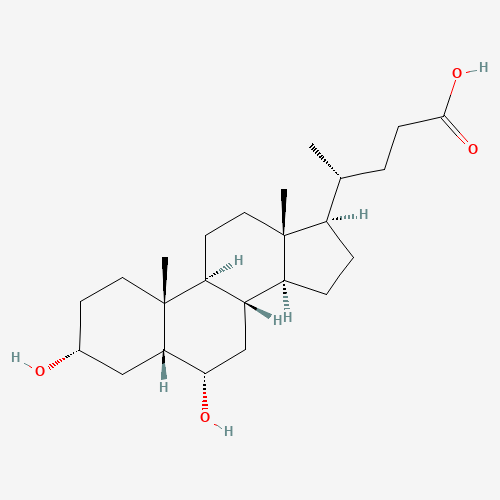 |
| 39 | 6-Gingerol | Phenol | C17H26O4 | 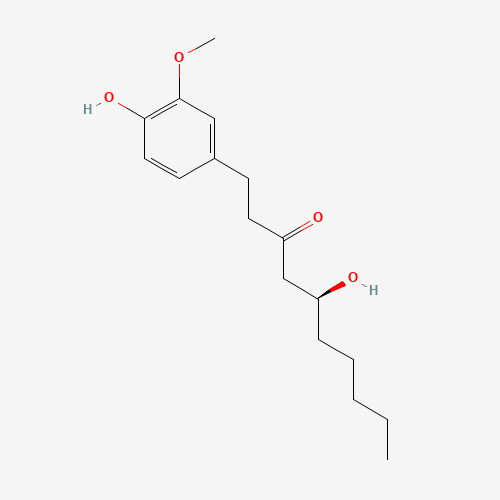 |
| 40 | Embelin | Quinones | C17H26O4 | 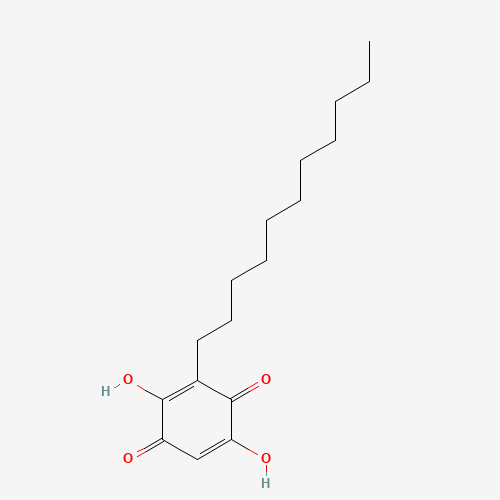 |
| 41 | Deoxycholic acid | Steroids | C24H40O4 | 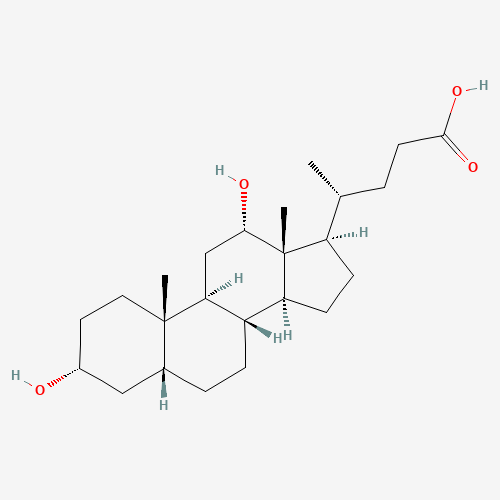 |
| 42 | Ginkgolic acid (C13:0) | Phenolic_acids | C20H32O3 | 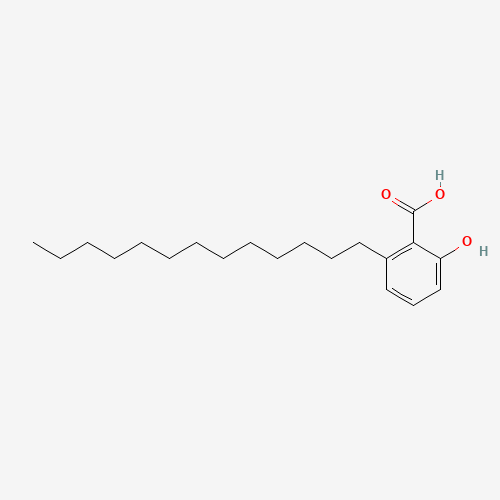 |
| 43 | Dihydroartemisinic acid | Terpenoids | C15H24O2 | 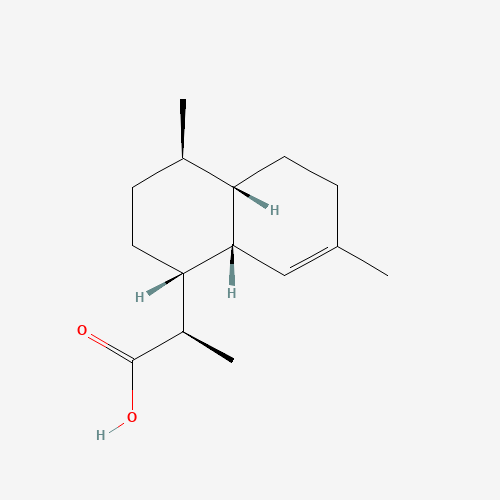 |
| 44 | Pristimerin | Terpenoids | C30H40O4 | 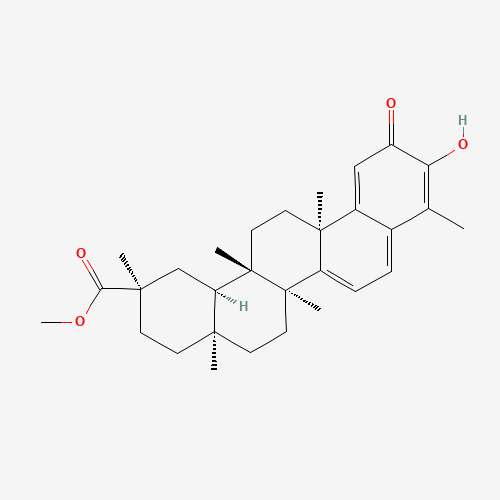 |
| 45 | 8-Desoxygartanin | Flavonoids | C23H24O5 | 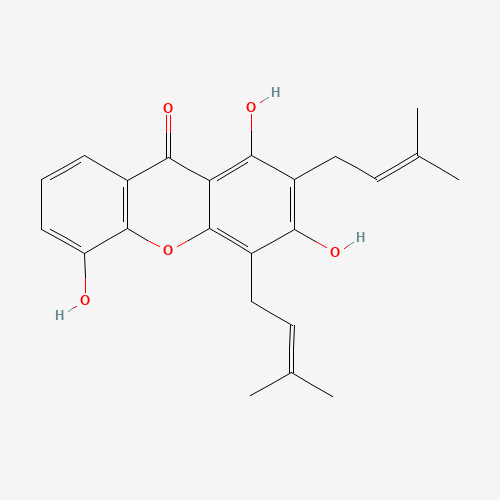 |
| 46 | Citric acid | Miscellaneous | C6H8O7 | 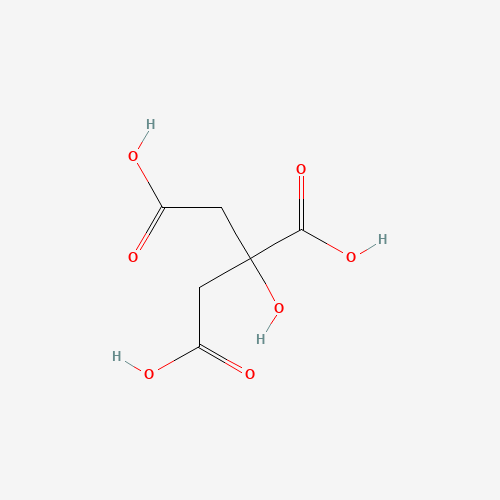 |

**Supplementary 2.**

**Supplementary 2.1**

| **Term** | **FDR** | **PValue** | **Count** | **Fold Enrichment** |
| --- | --- | --- | --- | --- |
| GO:0007165~signal transduction | 7.18E-11 | 4.6E-13 | 44 | 3.514438 |
| GO:0043066~negative regulation of apoptotic process | 5.17E-19 | 2.07E-22 | 39 | 7.529044 |
| GO:0006915~apoptotic process | 4.1E-14 | 8.2E-17 | 35 | 5.906781 |
| GO:0006468~protein phosphorylation | 3.72E-17 | 2.98E-20 | 34 | 7.994539 |
| GO:0045944~positive regulation of transcription from RNA polymerase II promoter | 3.33E-06 | 5.73E-08 | 34 | 2.887638 |
| GO:0006508~proteolysis | 1.21E-12 | 4.35E-15 | 29 | 6.625323 |
| GO:0008284~positive regulation of cell proliferation | 5.38E-11 | 3.23E-13 | 29 | 5.567702 |
| GO:0030335~positive regulation of cell migration | 5.41E-15 | 6.5E-18 | 26 | 10.18912 |
| GO:0009410~response to xenobiotic stimulus | 9.77E-15 | 1.57E-17 | 26 | 9.82128 |
| GO:0043065~positive regulation of apoptotic process | 3.14E-12 | 1.64E-14 | 25 | 7.762196 |

**Supplementary 2.1** The top 10 results of BP from GO enrichment analysis.

**Supplementary 2.2**

| **Term** | **FDR** | **PValue** | **Count** | **Fold Enrichment** |
| --- | --- | --- | --- | --- |
| GO:0005829~cytosol | 2.18E-18 | 2.26E-20 | 111 | 2.190328 |
| GO:0005737~cytoplasm | 1.2E-12 | 2.5E-14 | 102 | 1.954741 |
| GO:0005634~nucleus | 3.08E-08 | 9.59E-10 | 95 | 1.728161 |
| GO:0005886~plasma membrane | 1.62E-07 | 7.82E-09 | 86 | 1.750178 |
| GO:0005576~extracellular region | 9.41E-24 | 3.26E-26 | 76 | 3.814605 |
| GO:0005615~extracellular space | 9.11E-21 | 6.3E-23 | 68 | 3.797445 |
| GO:0005654~nucleoplasm | 0.000331 | 2.86E-05 | 61 | 1.661906 |
| GO:0070062~extracellular exosome | 5.15E-13 | 7.12E-15 | 60 | 2.97523 |
| GO:0005739~mitochondrion | 1.62E-07 | 7.83E-09 | 38 | 2.877205 |
| GO:0032991~macromolecular complex | 7.19E-09 | 1.74E-10 | 28 | 4.430353 |

**Supplementary 2.2** The top 10 results of CC from GO enrichment analysis.

**Supplementary 2.3**

| **Term** | **FDR** | **PValue** | **Count** | **Fold Enrichment** |
| --- | --- | --- | --- | --- |
| GO:0005515~protein binding | 3.27E-09 | 6.63E-11 | 162 | 1.312856 |
| GO:0042802~identical protein binding | 1.8E-17 | 6.63E-20 | 62 | 3.661211 |
| GO:0005524~ATP binding | 7.61E-10 | 1.26E-11 | 46 | 3.06188 |
| GO:0004712~protein serine/threonine/tyrosine kinase activity | 2.74E-19 | 5.06E-22 | 36 | 8.298038 |
| GO:0019899~enzyme binding | 1.23E-14 | 9.08E-17 | 29 | 7.666537 |
| GO:0008270~zinc ion binding | 1.22E-06 | 3.6E-08 | 29 | 3.346025 |
| GO:0042803~protein homodimerization activity | 6.55E-07 | 1.69E-08 | 27 | 3.697557 |
| GO:0004672~protein kinase activity | 4.15E-12 | 5.36E-14 | 26 | 6.945045 |
| GO:0019901~protein kinase binding | 1.27E-06 | 3.99E-08 | 22 | 4.314733 |
| GO:0004713~protein tyrosine kinase activity | 3.41E-16 | 1.89E-18 | 20 | 17.99526 |

**Supplementary 2.3** The top 10 results of MF from GO enrichment analysis.

**Supplementary 3.**

| **Term** | **FDR** | **PValue** | **Count** | **Fold Enrichment** |
| --- | --- | --- | --- | --- |
| hsa05200:Pathways in cancer | 8.55E-33 | 6.57E-35 | 66 | 6.098208 |
| hsa04151:PI3K-Akt signaling pathway | 2.66E-16 | 1.02E-17 | 39 | 5.329948 |
| hsa05417:Lipid and atherosclerosis | 7.5E-23 | 1.15E-24 | 38 | 8.671575 |
| hsa04010:MAPK signaling pathway | 1.48E-12 | 2.5E-13 | 31 | 5.052985 |
| hsa05205:Proteoglycans in cancer | 8.41E-16 | 5.18E-17 | 30 | 7.17993 |
| hsa04014:Ras signaling pathway | 1.73E-13 | 2.13E-14 | 29 | 6.02891 |
| hsa05161:Hepatitis B | 1.76E-14 | 1.22E-15 | 26 | 7.874286 |
| hsa04015:Rap1 signaling pathway | 3.16E-12 | 5.62E-13 | 26 | 6.074449 |
| hsa04115:p53 signaling pathway | 1.41E-21 | 3.26E-23 | 25 | 16.57529 |
| hsa05215:Prostate cancer | 1.39E-18 | 4.28E-20 | 25 | 12.64507 |
| hsa05208:Chemical carcinogenesis - reactive oxygen species | 5.86E-11 | 1.53E-11 | 25 | 5.50032 |
| hsa04210:Apoptosis | 3.07E-14 | 2.37E-15 | 24 | 8.658151 |
| hsa05162:Measles | 3.92E-14 | 3.31E-15 | 24 | 8.532671 |
| hsa05152:Tuberculosis | 5.96E-12 | 1.15E-12 | 24 | 6.541714 |
| hsa05169:Epstein-Barr virus infection | 5.34E-11 | 1.31E-11 | 24 | 5.82925 |
| hsa04510:Focal adhesion | 5.74E-11 | 1.46E-11 | 24 | 5.800535 |
| hsa04068:FoxO signaling pathway | 1.07E-13 | 1.15E-14 | 23 | 8.614089 |
| hsa05418:Fluid shear stress and atherosclerosis | 2.84E-13 | 4.15E-14 | 23 | 8.118314 |
| hsa05225:Hepatocellular carcinoma | 1.07E-11 | 2.23E-12 | 23 | 6.716939 |
| hsa05207:Chemical carcinogenesis - receptor activation | 7.59E-10 | 2.37E-10 | 23 | 5.322857 |

**Supplementary 3.** The top 20 pathways based on KEGG enrichment analysis .
